# Convergent Evolution of Hemoglobin Function in High-Altitude Andean Waterfowl Involves Limited Parallelism at the Molecular Sequence Level

**DOI:** 10.1371/journal.pgen.1005681

**Published:** 2015-12-04

**Authors:** Chandrasekhar Natarajan, Joana Projecto-Garcia, Hideaki Moriyama, Roy E. Weber, Violeta Muñoz-Fuentes, Andy J. Green, Cecilia Kopuchian, Pablo L. Tubaro, Luis Alza, Mariana Bulgarella, Matthew M. Smith, Robert E. Wilson, Angela Fago, Kevin G. McCracken, Jay F. Storz

**Affiliations:** 1 School of Biological Sciences, University of Nebraska, Lincoln, Nebraska, United States of America; 2 Department of Bioscience, Zoophysiology, Aarhus University, Aarhus, Denmark; 3 Estación Biológica de Doñana-CSIC, Sevilla, Spain; 4 Conservation Genetics Group, Senckenberg Research Institute and Natural History Museum Frankfurt, Gelnhausen, Germany; 5 Centro de Ecología Aplicada del Litoral (CECOAL), Consejo Nacional de Investigaciones Cientificas y Técnicas (CONICET), Corrientes, Argentina; 6 División Ornitología, Museo Argentino de Ciencias Naturales ‘Bernardino Rivadavia’ (MACN-CONICET), Buenos Aires, Argentina; 7 Institute of Arctic Biology and University of Alaska Museum, University of Alaska Fairbanks, Fairbanks, Alaska, United States of America; 8 Department of Biology and Department of Marine Biology and Ecology, Rosenstiel School of Marine and Atmospheric Sciences, University of Miami, Coral Gables, Florida, United States of America; University of California Davis, UNITED STATES

## Abstract

A fundamental question in evolutionary genetics concerns the extent to which adaptive phenotypic convergence is attributable to convergent or parallel changes at the molecular sequence level. Here we report a comparative analysis of hemoglobin (Hb) function in eight phylogenetically replicated pairs of high- and low-altitude waterfowl taxa to test for convergence in the oxygenation properties of Hb, and to assess the extent to which convergence in biochemical phenotype is attributable to repeated amino acid replacements. Functional experiments on native Hb variants and protein engineering experiments based on site-directed mutagenesis revealed the phenotypic effects of specific amino acid replacements that were responsible for convergent increases in Hb-O_2_ affinity in multiple high-altitude taxa. In six of the eight taxon pairs, high-altitude taxa evolved derived increases in Hb-O_2_ affinity that were caused by a combination of unique replacements, parallel replacements (involving identical-by-state variants with independent mutational origins in different lineages), and collateral replacements (involving shared, identical-by-descent variants derived via introgressive hybridization). In genome scans of nucleotide differentiation involving high- and low-altitude populations of three separate species, function-altering amino acid polymorphisms in the globin genes emerged as highly significant outliers, providing independent evidence for adaptive divergence in Hb function. The experimental results demonstrate that convergent changes in protein function can occur through multiple historical paths, and can involve multiple possible mutations. Most cases of convergence in Hb function did not involve parallel substitutions and most parallel substitutions did not affect Hb-O_2_ affinity, indicating that the repeatability of phenotypic evolution does not require parallelism at the molecular level.

## Author Summary

The convergent evolution of similar traits in different species could be due to repeated changes at the genetic level or different changes that produce the same phenotypic effect. To investigate the extent to which convergence in phenotype is caused by repeated mutations, we investigated the molecular basis of convergent changes in the oxygenation properties of hemoglobin (Hb) in eight pairs of high- and low-altitude waterfowl taxa from the Andes. The results revealed that convergent increases in Hb-O_2_ affinity in highland taxa involved a combination of unique and repeated amino acid replacements. However, convergent changes in Hb function generally did not involve parallel substitutions, indicating that repeatability in the evolution of protein function does not require repeatability at the sequence level.

## Introduction

When multiple species evolve similar changes in phenotype in response to a shared environmental challenge, it suggests that the convergently evolved character state is adaptive under the changed conditions and that it evolved under the influence of directional selection. A key question in evolutionary genetics concerns the extent to which such cases of phenotypic convergence are caused by convergent or parallel substitutions in the underlying genes. This question has important implications for understanding the inherent repeatability of evolution at the molecular level [1–9].

In principle, the convergent evolution of a given phenotype may be attributable to (*i*) unique substitutions, (*ii*) parallel substitutions (where identical-by-state alleles with independent mutational origins fix independently in different lineages), or (*iii*) collateral substitutions (where shared, identical-by-descent alleles fix independently in different lineages)[8]. In the last case, allele-sharing between species may be due to the retention of ancestral polymorphism or a history of introgressive hybridization—either way, the function-altering alleles that contribute to phenotypic convergence do not have independent mutational origins.

One especially powerful means of assessing the pervasiveness of repeated evolution at the sequence level is to exploit natural experiments where phylogenetically replicated changes in protein function have evolved in multiple taxa as an adaptive response to a shared environmental challenge. For example, there are good reasons to expect that vertebrate species living at very high altitudes will have convergently evolved hemoglobins (Hbs) with increased O_2_-binding affinities [10,11]. Under severe hypoxia, an increased blood-O_2_ affinity can help ensure tissue O_2_ supply by safeguarding arterial O_2_ saturation while simultaneously maintaining the pressure gradient that drives O_2_ diffusion from the peripheral capillaries to the cells of respiring tissues [12–18]. Evolutionary adjustments in blood-O_2_ affinity often stem directly from structural changes in the tetrameric (α_2_β_2_) Hb protein. Genetically based changes in the oxygenation properties of Hb can be brought about by amino acid mutations that increase intrinsic Hb-O_2_ affinity and/or mutations that suppress the sensitivity of Hb to the inhibitory effects of allosteric co-factors in the red blood cell [19–22] (S1 Fig).

Derived increases in Hb-O_2_ affinity have been documented in some high-altitude birds and mammals [23–30], but other comparative studies have not revealed consistent trends [31–34]. Additional comparisons between conspecific populations and closely related species are needed to assess the validity of empirical generalizations about the relationship between Hb-O_2_ affinity and native elevation in vertebrates.

Previous surveys of sequence variation in the globin genes of Andean waterfowl documented repeated amino acid substitutions in the major Hb isoforms of multiple high-altitude taxa [35,36], but the functional effects of the substitutions were not assessed so it was not known whether the repeated changes contributed to convergent changes in the oxygenation properties of Hb. Here we report a comparative analysis of Hb function in eight phylogenetically replicated pairs of high- and low-altitude waterfowl taxa to test for convergent changes in biochemical phenotype, and to assess the extent to which convergent changes in phenotype are attributable to repeated amino acid substitutions. We measured the functional properties of native Hb variants in each population and species, and we used protein engineering experiments based on site-directed mutagenesis to measure the functional effects of repeated substitutions that were implicated in convergent increases in Hb-O_2_ affinity in high-altitude taxa. In six of the eight taxon pairs, the high-altitude taxa evolved derived increases in Hb-O_2_ affinity that were caused by a combination of unique, parallel, and collateral amino acid replacements. In comparisons involving high- and low-altitude populations of three different species, function-altering amino acid polymorphisms emerged as highly significant outliers in genome scans of nucleotide differentiation, with derived, affinity-enhancing mutations present at high frequency in the high-altitude populations. In combination with results of the functional experiments, the population genomic analyses provide an independent line of evidence that the observed changes in Hb function are attributable to positive directional selection.

## Results/Discussion

We examined differences in the structural and functional properties of the two adult-expressed Hb isoforms (HbA and HbD) from eight pairs of high-and low-altitude sister taxa. Two of the taxon pairs include sister species with contrasting elevational ranges: Andean goose (*Chloephaga melanoptera*)/Orinoco goose (*Neochen jubata*) and Puna teal (*Anas puna*)/silver teal (*A*. *versicolor*). The remaining six taxon pairs include high- and low-altitude populations of the same species: ruddy ducks (*Oxyura jamaicensis*), torrent ducks (*Merganetta armata*), crested ducks (*Lophonetta specularioides*), cinnamon teal (*Anas cyanoptera*), yellow-billed pintails (*Anas georgica*), and speckled teal (*Anas flavirostris*). In addition to the eight high- and low-altitude taxon pairs from the Andes, we also examined Hb function in a pair of high- and low-altitude sister species from Africa: the Abyssinian blue-winged goose (*Cyanochen cyanoptera*), a high-altitude species endemic to the Ethiopian Plateau, and Hartlaub’s duck (*Pteronetta hartlaubi*), a strictly lowland species [37]. We included these species in the analysis because their Hbs are distinguished by two amino acid replacements that are shared with multiple Andean taxa [35], so experimental tests of Hb function provide an additional opportunity to measure the functional effects of repeated substitutions.

### Hb Isoform Composition

To characterize the red cell Hb isoform composition of each species, we analyzed blood samples from individual specimens using a combination of isoelectric focusing (IEF) and tandem mass spectrometry (MS/MS). Consistent with data from other anseriform birds [38,39], the waterfowl species that we examined expressed two distinct isoforms, HbA (pI = 8.0–8.2) and HbD (pI = 7.0–7.2) with the major HbA isoform comprising ~70–80% of total Hb (S1 Table). The major HbA isoform incorporates α-chain products of the α^*A*^-globin gene and the minor HbD isoform incorporates products of the tandemly linked α^*D*^-globin gene; both isoforms incorporate β-chain products of the same β^*A*^-globin gene [38,39]. Since avian HbD has a consistently higher O_2_-affinity than HbA in all avian taxa examined to date [28,30,32,39], upregulating HbD expression could be expected to provide an efficient means of increasing blood-O_2_ affinity in response to environmental hypoxia. However, it appears that high-altitude Andean waterfowl do not avail themselves of this option, as we observed no difference in relative isoform abundance between pairs of high- and low-altitude sister taxa (Wilcoxon signed-rank test, *W* = 12, *N* = 7 pairwise comparisons, *P*>0.05; S1 Table). MS/MS analysis confirmed that subunits of the two adult Hb isoforms represent products of the adult-expressed α^*A*^-, α^*D*^-, and β^*A*^-globin genes; products of the embryonic α- and β-type globin genes were not detected.

### Hbs of High- and Low-Altitude Taxa are Distinguished by a Combination of Unique and Repeated Amino Acid Replacements

**Fig 1:**
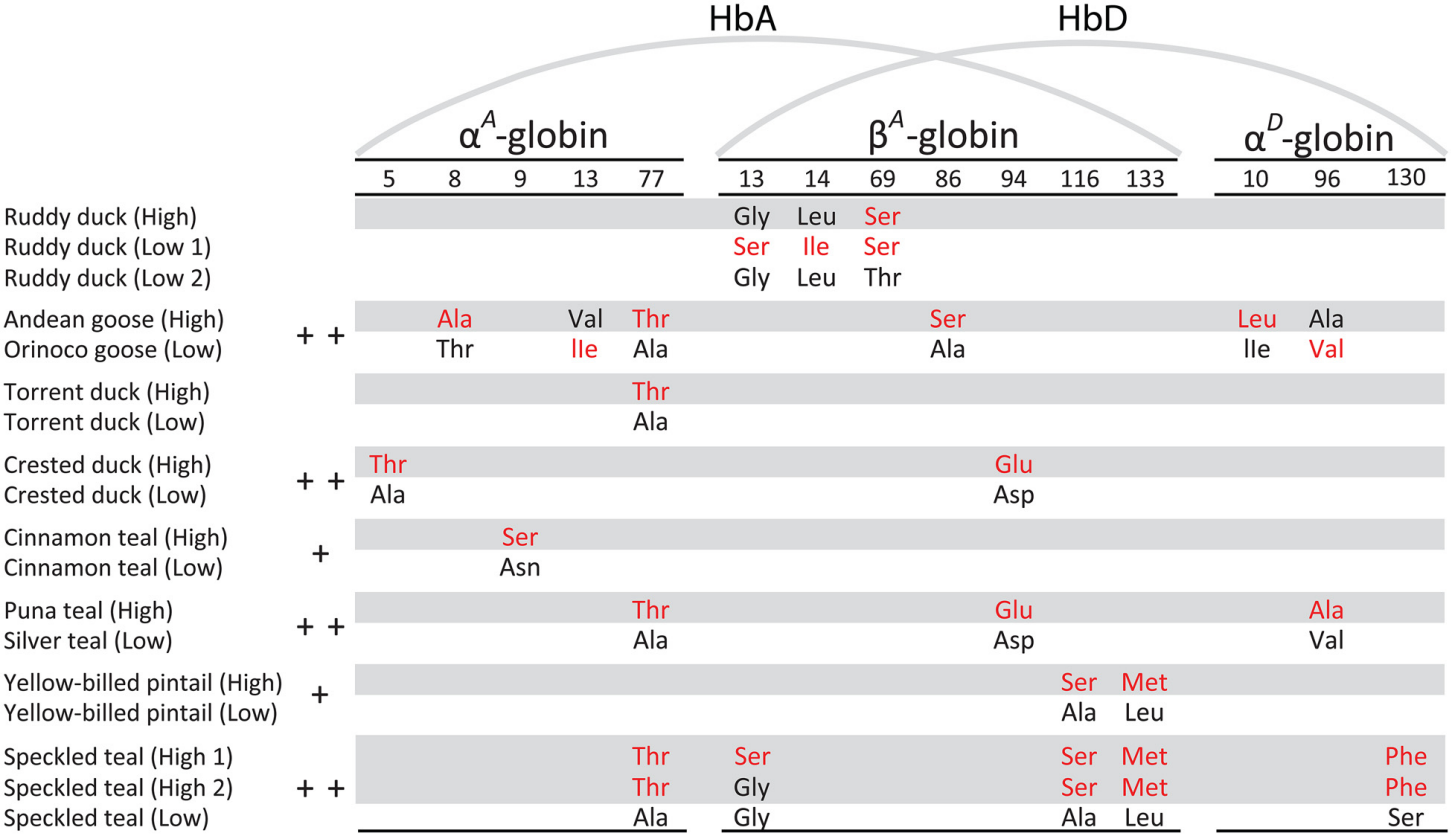
Amino acid differences that distinguish the HbA and HbD isoforms of each pair of high- and low-altitude sister taxa. Derived (non-ancestral) amino acids are shown in red lettering. Subunits of the major HbA isoform are encoded by the α^*A*^- and β^*A*^-globin genes, whereas those of the minor HbD isoform are encoded by the α^*D*^- and β^*A*^-globin genes. For each pair of high- and low-altitude sister taxa, ‘+’ indicates that HbA O_2_-affinity in the presence of allosteric effectors is significantly higher in the high-altitude taxon, and ‘++’ indicates that the O_2_-affinities of HbA and HbD are both significantly higher in the high-altitude taxon. Due to allelic polymorphism in the β^*A*^-globin gene, alternative HbA and HbD variants were present in the low-altitude sample of ruddy ducks (‘low1’ and ‘low2’) and in the high-altitude sample of speckled teal (‘high1’ and ‘high2’). The basis for inferring the direction of character-state change for each amino acid site is explained in S3 Fig (α^*A*^), S4 Fig (β^*A*^), and S5 Fig (α^*D*^).

By combining α^*D*^-globin sequences with previously published α^*A*^- and β^*A*^-globin sequences for the same individual specimens, we identified all amino acid differences that distinguish the HbA and HbD isoforms of each pair of high- and low-altitude taxa (Fig 1). Full alignments of α^*A*^-, α^*D*^-, and β^*A*^-globin amino acid sequences are shown in S2 Fig, and the direction of changes in character state at all substituted sites are shown in S3–S5 Figs. Comparisons of the South American species revealed repeated amino acid replacements at five sites that distinguish the HbA isoforms of high- and low-altitude sister taxa, including repeated replacements at one site in the α^*A*^-globin gene (α77Ala→Thr in Andean goose, torrent duck, Puna teal, and speckled teal) and four sites in the β^*A*^-globin gene (β13Gly→Ser in ruddy ducks and speckled teal, β94Asp→Glu in crested duck and Puna teal, and both β116Ala→Ser and β133Leu→Met in yellow-billed pintail and speckled teal)(Fig 1).

The derived pair of β^*A*^-globin amino acid variants ‘116Ser-133Met’ that are shared between sympatric high-altitude populations of yellow-billed pintails and speckled teal are clearly identical-by-descent (S6 Fig). Independent evidence for hybridization between the two species [40,41] suggests that the ‘116Ser-133Met’ β^*A*^-globin allele in high-altitude yellow-billed pintails was derived via introgression from high-altitude speckled teals. The same is true for a shared β13(Gly/Ser) polymorphism, although the derived Ser variant is present at low-frequency in yellow-billed pintails. The repeated amino acid changes at β^*A*^-globin sites 13, 116 and 133 therefore represent collateral replacements, rather than true parallel replacements, as they do not have independent mutational origins in each species.

Three of the eight pairs of high- and low-altitude taxa had structurally distinct HbD isoforms due to 1–2 amino acid substitutions in the α^*D*^-globin gene (Fig 1). Repeated substitutions at α^*D*^96 occurred in Orinoco goose (Val→Ala) and silver teal (Ala→Val), but the direction of the change in character-state was different in each case (S5 Fig). In both interspecific comparisons (Andean goose vs. Orinoco goose, and Puna teal vs. silver teal), α^*D*^96Ala is associated with a higher HbD O_2_-affinity. However, the individual effects of amino acid replacements at α^*D*^96 could not be isolated in either comparison because of potentially confounding replacements in the β-chain (β86Ala→Ser in Andean goose, and β94Asp→Glu in Puna teal)(Fig 1).

### Convergent Increases in Hb-O_2_ Affinity in High-Altitude Taxa

We measured the O_2_-binding properties of purified HbA and HbD variants from each taxon and we estimated *P*_50_ (the *P*O_2_ at which Hb is half-saturated with O_2_) as an index of Hb-O_2_ affinity. We focus primarily on measures of Hb-O_2_ affinity in the presence of Cl^-^ ions and IHP (*P*_50(KCl+IHP)_), as this is the experimental treatment that is most relevant to *in vivo* conditions in avian red blood cells. The experiments revealed that O_2_-affinities of HbD were consistently higher (*P*_50_ values were lower; S2 Table) than those of HbA, consistent with data from other birds [28,30,32,39].

**Fig 2:**
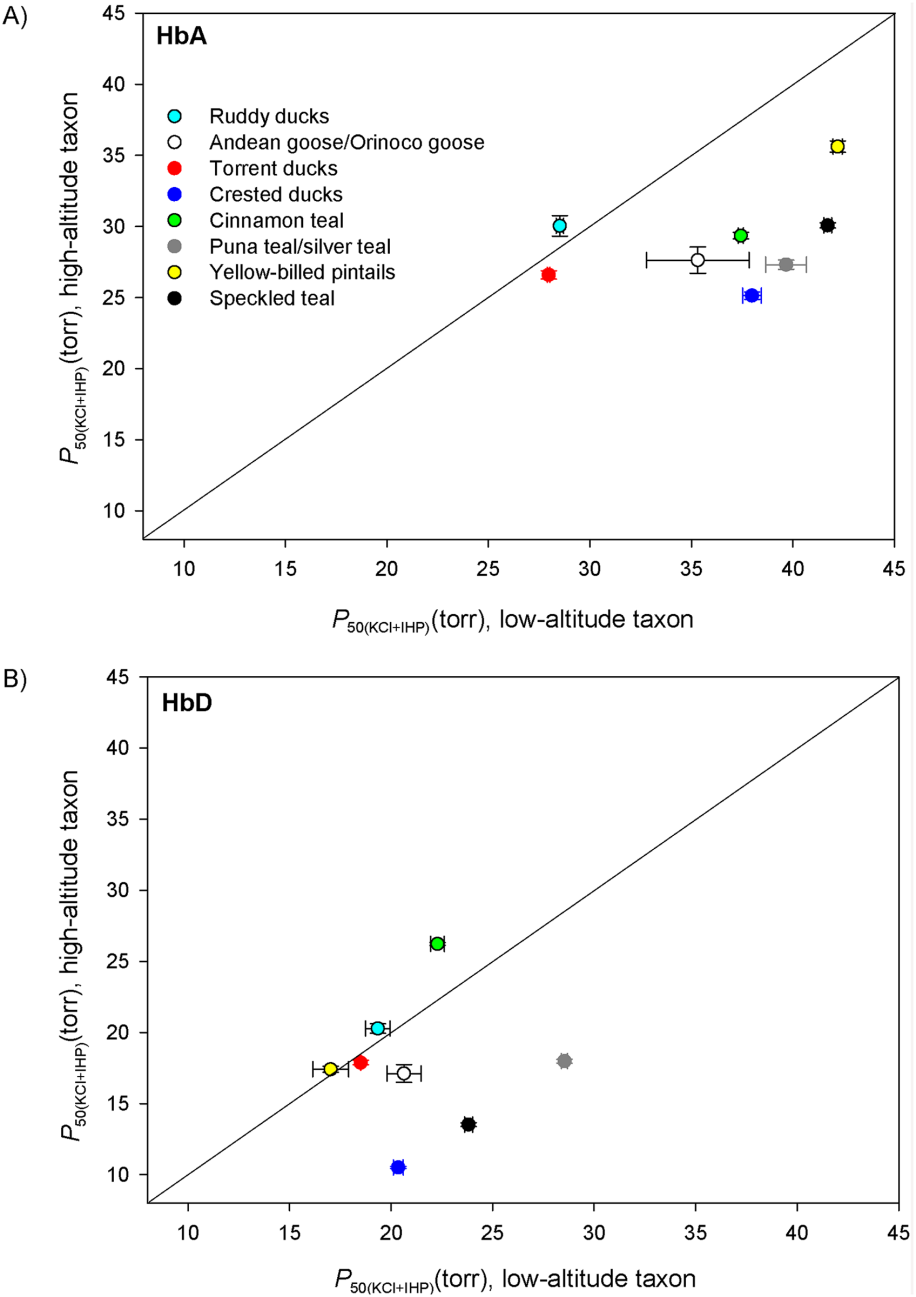
Convergent evolution of increased Hb-O_2_ affinities in high-altitude Andean waterfowl. (*A*) Plot of *P*_50(KCl+IHP)_ (mean ± SEM)(an inverse measure of Hb-O_2_ affinity in the presence of allosteric effectors) for the major HbA isoform in eight pairs of high- and low-altitude sister taxa. Data points that fall below the diagonal denote cases in which the high-altitude member of a given taxon pair possesses a higher Hb-O_2_ affinity (lower *P*_50_). (*B*) Plot of *P*_50(KCl+IHP)_ for the minor HbD isoform in the same eight taxon pairs.

Comparisons between high- and low-altitude sister taxa revealed appreciable differences in the O_2_-affinity of the major HbA isoform in six of eight cases, and in each of these six cases the HbA of the high-altitude taxon exhibited the higher O_2_-affinity (i.e., lower *P*_50_)(Fig 2A; S2 Table). The only two taxon pairs that did not exhibit appreciable differences in Hb-O_2_ affinity were those involving conspecific populations of ruddy ducks and torrent ducks (Fig 2A; S2 Table). In contrast to the altitudinal trend for HbA, O_2_-affinities of the minor HbD isoform were not consistently higher in high-altitude taxa (Fig 2B). However, there were three taxon pairs in which O_2_-affinities of HbA and HbD were both markedly higher in the high-altitude taxa than in the corresponding low-altitude taxa (crested duck, Puna teal, and speckled teal), a pattern that implicates causative mutations in the β-chain subunit, which is shared by both isoforms.

### Identification of Causal Mutations

Comparisons involving purified Hb variants from birds with known genotypes provide a means of identifying the specific amino acid mutations that are responsible for evolved changes in Hb-O_2_ affinity. Below we describe the functional effects of unique and repeated replacements, and we report model-based inferences about the structural mechanisms responsible for the observed changes in Hb-O_2_ affinity.

#### Unique substitutions

**Fig 3:**
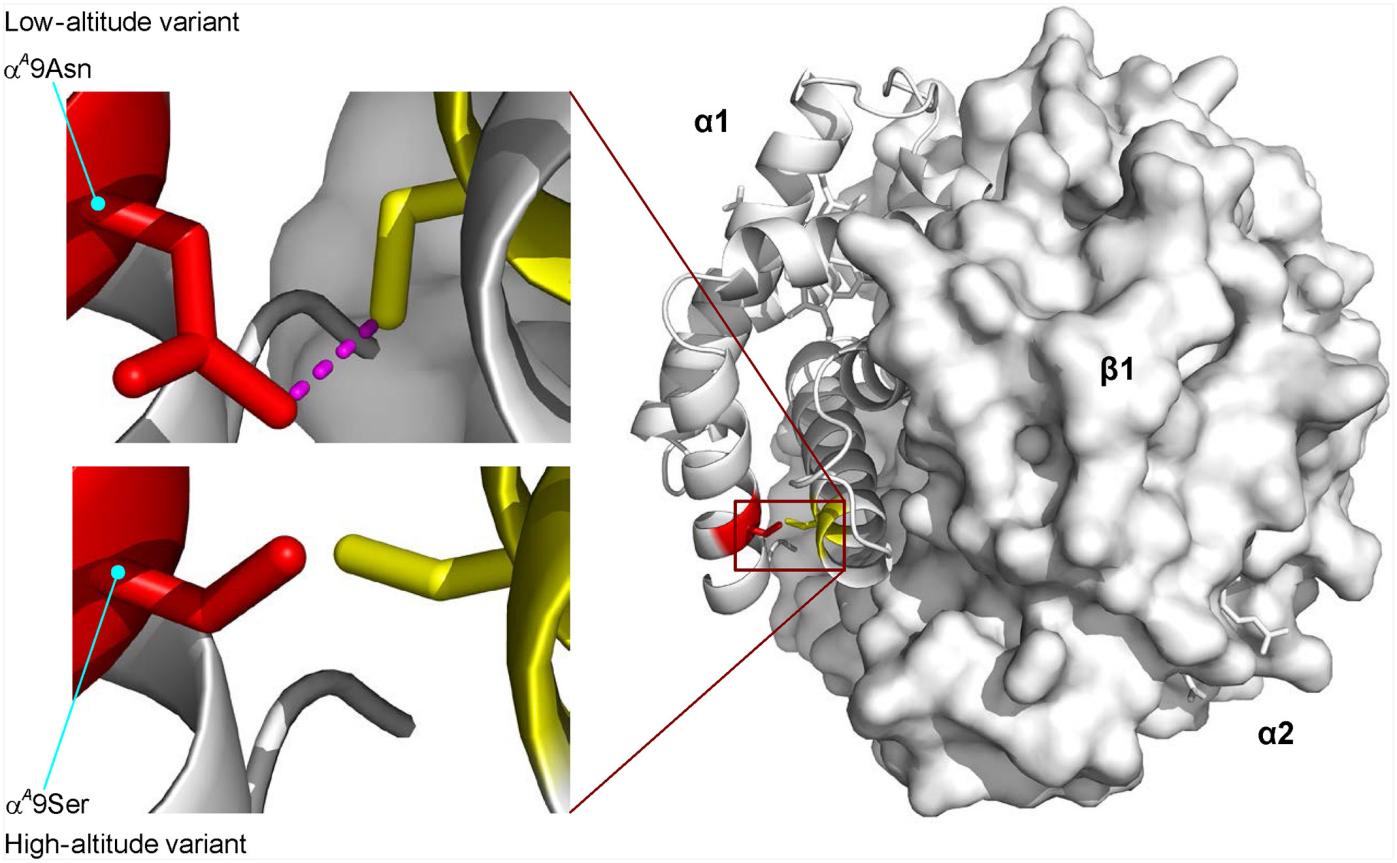
Homology model of cinnamon teal HbA showing the location of a single α-chain amino acid replacement (α^*A*^9Asn→Ser) that distinguishes high- and low-altitude variants. Replacement of the ancestral Asn with Ser at α^*A*^9 eliminates an intrasubunit hydrogen bond (shown in magenta) between the δ2-nitrogen of α^*A*^9Asn and the γ-oxygen of α^*A*^124Ser in deoxyHb. The loss of this noncovalent bond is predicted to destabilize the low-affinity T-state quaternary structure, thereby shifting the allosteric equilibrium in favor of the high-affinity R-state.

A single amino acid replacement (α^*A*^9Asn→Ser) distinguishes the HbA variants of high- and low-altitude cinnamon teal, and is associated with a 22% reduction in *P*_50(KCl+IHP)_ (an increase in O_2_ affinity) in the high-altitude α^*A*^9Ser Hb variant. This difference is exclusively attributable to a change in intrinsic O_2_-affinity, as the alternative HbA variants exhibit no difference in anion sensitivity (S7 Fig). Homology modeling revealed the apparent structural basis of the derived increase in Hb-O_2_ affinity: the replacement is predicted to eliminate the hydrogen bond between the δ2-nitrogen of α^*A*^9Asn and the γ-oxygen of α^*A*^124Ser in deoxy (T-state) Hb—the same bond is not present in the R-state conformation (Fig 3). This change is predicted to destabilize the low-affinity T-state quaternary structure, thereby shifting the allosteric equilibrium in favor of the high-affinity R-state.

**Fig 4:**
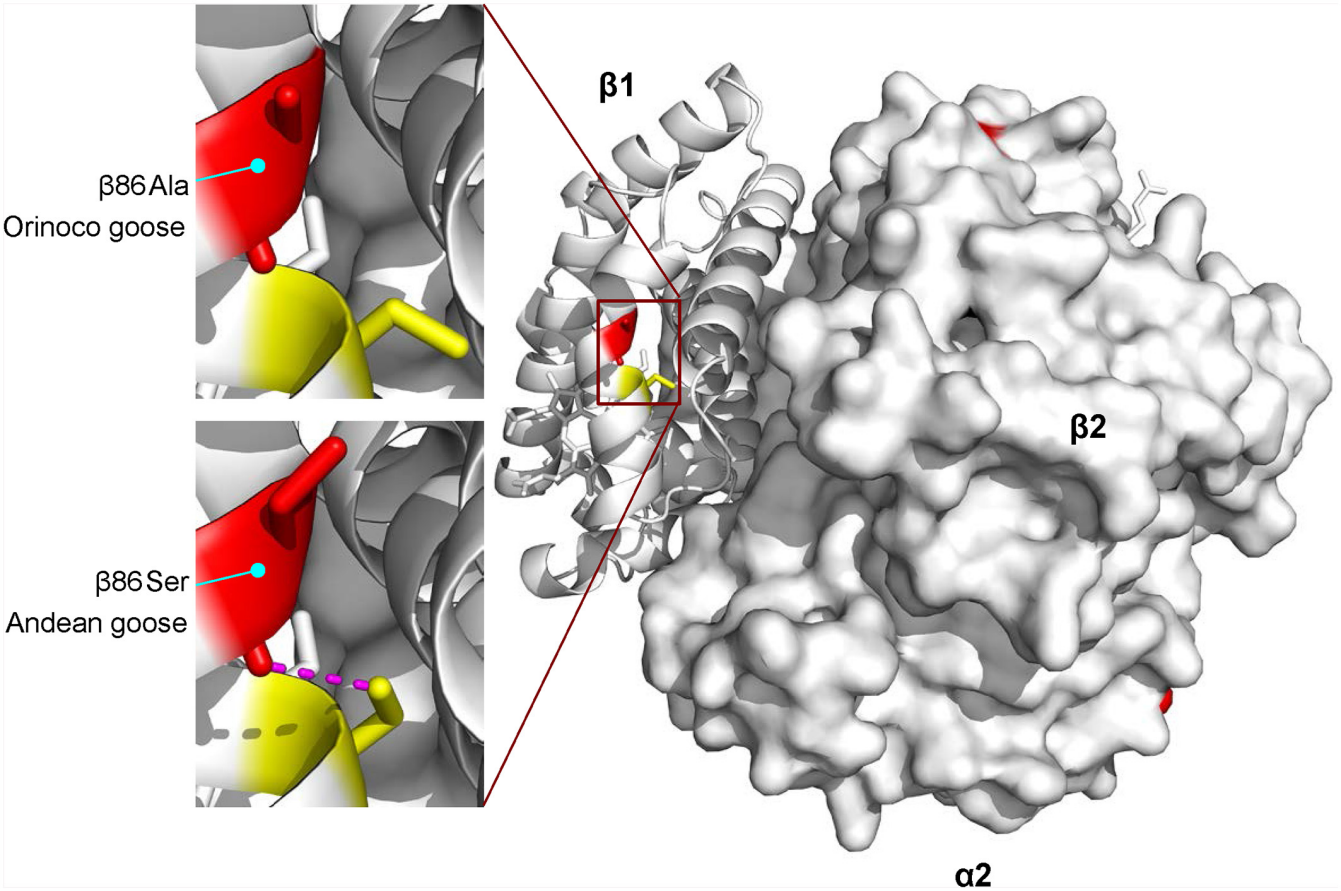
HbA isoforms of the high-altitude Andean goose and the low-altitude Orinoco goose are distinguished by four amino acid substitutions, one of which (β86Ala→Ser) appears to be mainly responsible for the observed species difference in intrinsic O_2_-affinity. Replacing the ancestral Ala with Ser at β86 (the 2^nd^ residue of the F-helix) results in the addition of a helix-capping hydrogen bond (shown in magenta) between the carbonyl oxygen of β86Ser and the γ-oxygen of β89Ser. The resultant stabilization of the F-helix is predicted to increase O_2_-affinity via subtle displacements of covalent and noncovalent β-heme contacts.

HbA isoforms of the high-altitude Andean goose and the low-altitude Orinoco goose differ at three sites in the α^*A*^-chain and one in the β-chain (Fig 1). The independent or joint effects of these four amino acid replacements account for a 22% reduction in *P*_50(KCl+IHP)_ of Andean goose HbA relative to that of Orinoco goose. Similar to the case with the cinnamon teal HbA variants, this difference is attributable to a change in intrinsic O_2_-affinity (S8 Fig). One of the α^*A*^-globin substitutions in Andean goose is shared with several other high-altitude taxa (α^*A*^77Ala→Thr), but for reasons described below, this substitution seems unlikely to contribute to the observed species difference in Hb-O_2_ affinity. Instead, homology-modeling suggests that the unique β86Ala→Ser replacement is primarily responsible for the increased O_2_-affinity of Andean goose Hb due to the formation of a helix-capping hydrogen bond between the carbonyl oxygen of β86Ser and the γ-oxygen of β89Ser (Fig 4)–a contact that stabilizes the F-helix. Mutations at β86 in human Hb also increase intrinsic O_2_-affinity by perturbing the tertiary structure of the F-helix and FG corner, resulting in a displacement of residues that contact the β-heme porphyrin ring [42,43].

#### Repeated substitutions that have no apparent effects on oxygenation properties of Hb

An examination of the two taxon pairs that exhibited no difference in HbA O_2_-affinity suggests that it may be possible to rule out causative effects for two of the five repeated substitutions. HbA variants of high- and low-altitude torrent ducks differ at a single site, α^*A*^77, which makes for an especially clean comparison. High-altitude torrent ducks share the same α^*A*^77Ala→Thr replacement with three other high-altitude Andean taxa (Andean goose, Puna teal, and speckled teal). This amino acid replacement has no effect on Hb-O_2_ affinity in torrent ducks (S2 Table), suggesting that the same replacement is unlikely to contribute to net changes in Hb-O_2_ affinity in the other species. Consistent with these results, a recent experimental study of Hb polymorphism in rufous-collared sparrows (*Zonotrichia capensis*) [32] documented that an Ala→Ser mutation at α^*A*^77 had no discernible effect on oxygenation properties of Hb (this mutation, like the α^*A*^77Ala→Thr mutation at the same site in waterfowl Hb, involves the replacement of nonpolar Ala for a residue with an uncharged, polar sidechain). Structural considerations suggest no reason to expect the α^*A*^77Ala→Thr mutation to affect O_2_-binding [32], but we cannot rule out the possibility that the mutation has different effects on the genetic backgrounds of different species.

Similar to the case of the torrent ducks, HbA and HbD variants of high- and low-altitude ruddy ducks differ at three β-chain sites (Fig 1), and a replacement at one of these sites (β13Gly→Ser) in low-altitude ruddy ducks is shared with high-altitude speckled teal. Since this amino acid replacement has no discernible net effect on the O_2_ affinities of HbA or HbD in ruddy ducks, it seems unlikely that the same replacement contributes to changes in Hb-O_2_ affinity in other species (although, again, we cannot rule out the possibility that the same mutation produces different effects on the genetic backgrounds of different species).

Interestingly, the parallel replacements at α^*A*^77 and β13 are both caused by nonsynonymous transition mutations at CpG dinucleotides. Since rates of transition mutation at CpG sites are roughly an order of magnitude higher than those at other nucleotide sites in mammalian and avian genomes [44–47], the recurrent changes at α^*A*^77 and β13 suggest that patterns of parallel substitution may be strongly influenced by mutation bias. Recurrent changes at both sites are also observed in a broader taxonomic sample of waterfowl taxa (S3 and S4 Figs).

#### Parallel replacements that contribute to convergence in Hb function

Parallel β94Asp→Glu replacements that occurred in high-altitude crested ducks and Puna teal are associated with pronounced increases in the O_2_-affinities of both HbA and HbD relative to variants of the same isoforms that predominate in low-altitude sister taxa (S9 and S10 Figs; S2 Table), although there is potentially confounding amino acid variation in the α^*A*^-globin gene (Fig 1). In the HbA isoforms of both species, β94Asp→Glu is associated with a slightly higher intrinsic O_2_-affinity (S9A and S9B Fig, S10A and S10B Fig), and in both HbA and HbD it is generally associated with a suppressed anion sensitivity (S9C and S9D Fig, S10C and S10D Fig). With regard to overall effects on Hb-O_2_ affinity, the β94Asp→Glu mutation appears to have similar affinity-enhancing effects on different genetic backgrounds, as revealed by comparisons between the same isoforms in different species (orthologous comparisons), and comparisons between different isoforms in the same and in different species (paralogous comparisons). The high- and low-altitude HbA variants of crested ducks exhibited a 1.5-fold difference in *P*_50(KCl+IHP)_ (25.14 vs. 37.98 torr, respectively), as did the HbA isoforms of Puna teal and silver teal (27.32 vs. 39.66 torr, respectively). The fact that the differences in *P*_50_ were identical in magnitude in both pairwise comparisons suggests that the shared β94Asp→Glu replacement in high-altitude crested ducks and Puna teal accounts for all or most of the observed difference in HbA O_2_-affinity, and that the additional α^*A*^5Ala→Thr replacement in high-altitude crested ducks is less consequential. Moreover, the comparison between HbD variants of crested ducks cleanly isolates the effect of the β94Asp→Glu mutation, as there is no confounding variation in the α^*D*^-globin gene (Fig 1). This single amino acid replacement produced a 1.9-fold reduction in *P*_50(KCl+IHP)_ (20.35 vs. 10.51 torr; S2 Table). The β94Asp→Glu mutation is therefore associated with significant increases in Hb-O_2_ affinity on all backgrounds in which it occurs.

In human Hb, mutational replacements of β94Asp increase O_2_-affinity and greatly diminish the pH-dependence of O_2_-binding (Bohr effect) by disrupting the salt bridge between the carboxyl group of β94Asp and the imidazole group of the C-terminal β146His of the same β subunit [48–50]. Elimination of this intra-chain salt bridge destabilizes the low-affinity T-state conformation of the Hb tetramer, thereby increasing Hb-O_2_ affinity by shifting the allosteric equilibrium in favor of the high-affinity R-state. Disruption of the electrostatic interaction between β94 and β146 greatly diminishes the Bohr effect by attenuating the charge stabilization of β146His [51]. In human Hb, the β94Asp→Glu mutation diminishes the Bohr effect by ~50%, and crystallographic analysis confirmed that the salt bridge is not formed between β94Glu and β146His [50].

Consistent with data for human β94 Hb mutants, the β94Asp→Glu mutation in crested ducks and Puna teal produces a significant increase in O_2_-affinity. However, in contrast to the case with the human Hb mutants, additional experiments on HbA variants of crested ducks and Puna teal revealed that the β94Asp→Glu mutation increased O_2_-affinity without compromising the Bohr effect: in both species, Bohr factors (Δlog-*P*_50_/ΔpH) were similar for HbA variants with and without the β94Asp→Glu mutation (Table 1). These results are consistent with crystallographic data for avian Hb [52], which reveal the absence of the salt bridge between the carbonyl group of β94Asp and the imidazole of β146His. This explains why neither β94Asp nor β146His make significant contributions to the Bohr effect in avian Hbs, in accordance with the view that the Bohr effect is attributable to different sets of solvent-exposed His residues in the Hbs of different vertebrate taxa [53,54]. The positive Bohr factors for the stripped HbA variants are typical of Hbs with high phosphate sensitivities. Under stripped conditions (in the absence of phosphates) at low pH, electrostatic repulsion between positively charged residues in the central cavity destabilizes the T-state and increases O_2_-affinity. These charges are neutralized in the IHP-Hb complex and the Bohr effect is normal.

**Table 1:** O_2_-affinities, anion sensitivities, and pH sensitivities of duck HbA isoforms with alternative residues at β94 (derived Glu in high-altitude taxa, and ancestral Asp in low-altitude taxa). O_2_-affinity was measured as *P*_50_ (torr, mean ± SEM), anion sensitivity was measured as the difference in log-transformed *P*_50_ values in the presence and absence of anionic effectors (Δlog *P*_50([KCl+IHP]—stripped)_), and pH sensitivity was measured by the Bohr factor (Φ = Δlog *P*_50_/ΔpH). All measurements were conducted at 37°C, pH 6.9–7.4 (as indicated) in 0.1 M HEPES buffer in the absence and simultaneous presence of Cl^-^ ions (0.1 M KCl) and IHP (at two-fold molar excess over tetrameric Hb); [Heme] = 0.300 mM.

Taxon	Property	pH 7.4	pH 6.9	Φ (6.9–7.4)
Crested duck HbA (high)	*P*_50(stripped)_	2.66 ± 0.02	2.66 ± 0.05	0.00
	*P*_50(KCl+IHP)_	25.14 ± 0.25	51.84 ± 0.73	-0.63
	Δlog *P*_50([KCl+IHP]—stripped)_	0.98	1.29	
Crested duck HbA (low)	*P*_50(stripped)_	3.45 ± 0.02	2.91 ± 0.00	+0.15
	*P*_50(KCl+IHP)_	37.98 ± 0.46	74.87 ± 2.11	-0.59
	Δlog *P*_50([KCl+IHP]—stripped)_	1.04	1.41	
Puna teal HbA (high)	*P*_50(stripped)_	3.38 ± 0.01	2.55 ± 0.04	+0.25
	*P*_50(KCl+IHP)_	27.32 ± 0.33	48.70 ± 1.17	-0.50
	Δlog *P*_50([KCl+IHP]—stripped)_	0.91	1.28	
Puna teal HbA (low)	*P*_50(stripped)_	3.93 ± 0.02	3.51 ± 0.04	+0.10
	*P*_50(KCl+IHP)_	39.66 ± 1.01	80.04 ± 0.37	-0.61
	Δlog *P*_50([KCl+IHP]—stripped)_	1.00	1.36	

In light of data for native and recombinant human Hbs with mutational replacements of β94Asp [48–50], the fact that the β94Asp→Glu mutation in duck Hb produced an increased O_2_-affinity without an associated diminution of the Bohr effect illustrates how specific mutations with well-characterized effects in the Hb of one species can have quite different biochemical effects in the Hbs of other species [31,55,56].

#### Collateral replacements that contribute to convergence in Hb function

High-altitude yellow-billed pintails and speckled teal share the same two β-globin replacements, β116Ala→Ser and β133Leu→Met, due to a history of introgressive hybridization (S6 Fig). The β116Ala→Ser replacement is also shared by the high-altitude blue-winged goose. The HbA variants of these three high-altitude taxa generally exhibit higher O_2_-affinities than those of their respective low-altitude sister taxa, both in the presence and absence of anionic effectors (S2 Table). Comparisons between HbD variants of yellow-billed pintails are also informative because the high- and low-altitude populations have identical α^*D*^-globin sequences, so the main HbD variants only differ at β116 and β133 (Fig 1). The HbA isoforms of blue-winged goose and Hartlaub’s duck are distinguished from one another by two amino acid substitutions: α^*A*^77Ala→Thr and β116Ala→Ser (character states at both sites are derived in the high-altitude blue-winged goose)(S3 and S4 Figs) [35]. The HbA and HbD variants of yellow-billed pintails and the HbA variant of blue-winged goose are characterized by increased intrinsic O_2_-affinities relative to the corresponding variants in their respective low-altitude sister taxa (S11 Fig; S2 Table). These differences in intrinsic O_2_-affinity persist in the presence of Cl^-^ ions and IHP, but not in the presence of IHP alone (in the comparison between the HbA isoforms of blue-winged goose and Hartlaub’s duck) or in the simultaneous presence of both anions (in the comparison between high- and low-altitude HbD variants of yellow-billed pintail; S11 Fig; S2 Table). These comparisons indicate that the β116Ala→Ser and β133Leu→Met substitutions produce an increased intrinsic O_2_-affinity, and the blue-winged goose/Hartlaub duck comparison further suggests that this net effect is primarily or exclusively attributable to β116Ala→Ser.

**Fig 5:**
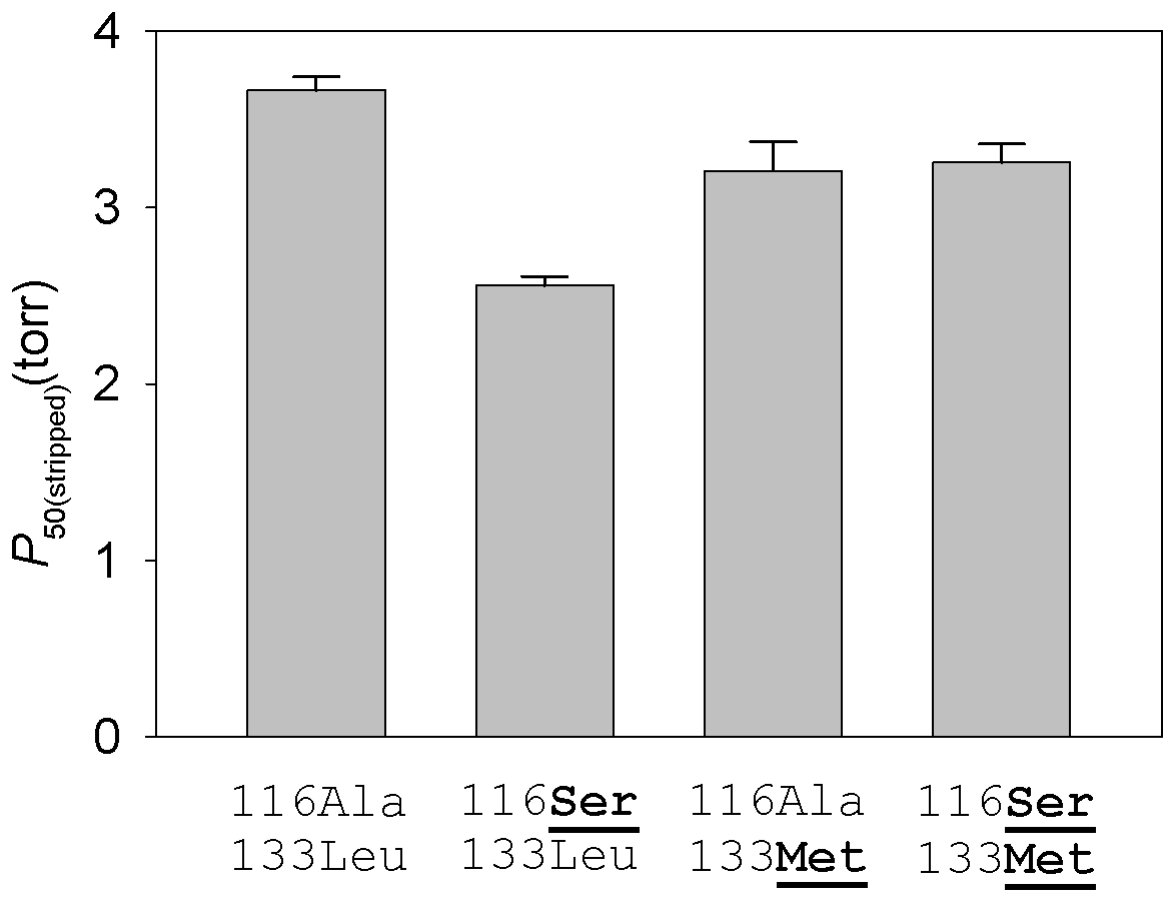
Intrinsic O_2_-affinities (*P*_50(stripped)_, torr; mean ± SEM) of purified yellow-billed pintail rHb mutants measured in the absence of allosteric effectors. O_2_-equilibrium curves for each rHb mutant were measured in 0.1 M HEPES buffer at pH 7.40, 37°C; [heme], 0.3 mM. Numbers refer to residue positions in the β-chain subunit. ‘116Ala-133Leu’ and ‘116Ser-133Met’ are the two-site genotypes that predominate in low- and high-altitude populations, respectively. At each site, the derived (non-ancestral) amino acids are underlined in bold.

To measure the independent and joint effects of the β116Ala→Ser and β133Leu→Met replacements on a standardized genetic background, we used site-directed mutagenesis to engineer recombinant yellow-billed pintail HbA mutants representing each of four possible two-site genotypic combinations: the wildtype low-altitude genotype (116Ala-133Leu, which represents the ancestral low-altitude state; S4 Fig), the derived, double-mutant high-altitude genotype (116Ser-133Met), and each of the two possible single-mutant intermediates (116Ser-133Leu and 116Ala-133Met). Experimental measurements of the rHb mutants recapitulated the observed difference in intrinsic O_2_-affinity between the HbA variants of high- and low-altitude yellow-billed pintails (*P*_50(stripped)_ [mean ± SEM] = 3.66 ± 0.08 torr for 116Ala-133Leu and 3.26 ± 0.10 torr for 116Ser-133Met), and revealed that the observed difference is mainly attributable to β116Ala→Ser (Fig 5). Relative to the ancestral background (116Ala-133Leu), β116Ala→Ser produced a 30% reduction in *P*_50(stripped)_, which translated into a 27% reduction in the presence of Cl^-^, a 10% reduction in the presence of IHP, and a 16% reduction in the simultaneous presence of both anionic effectors. The individual effect of β116Ala→Ser on the ancestral background is therefore consistent with inferences gleaned from comparisons involving the native HbA and HbD variants of yellow-billed pintails and the HbA isoforms of blue-winged goose and Hartlaub’s duck (S11 Fig).

**Fig 6:**
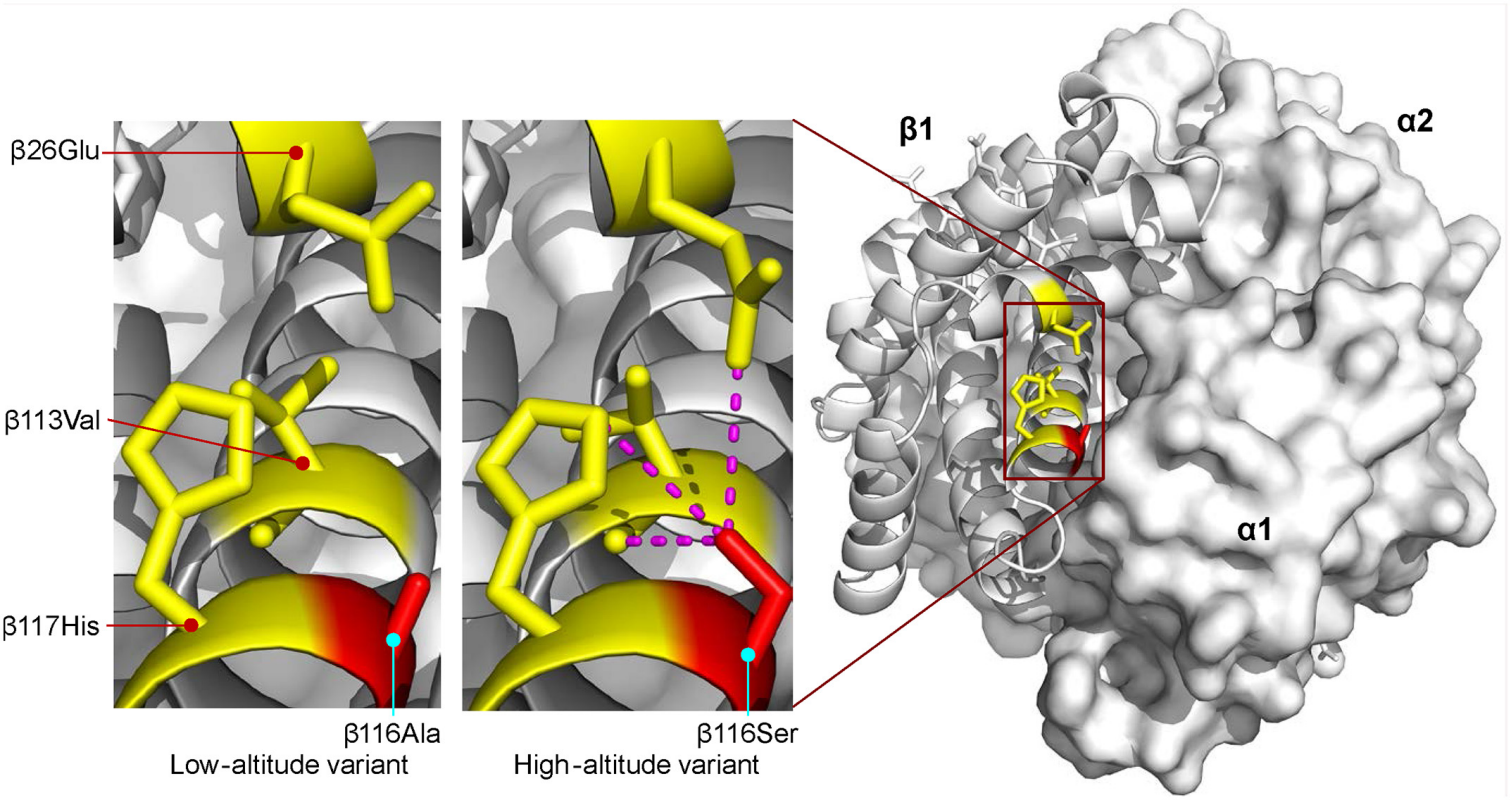
Homology model of yellow-billed pintail HbA showing the location of an affinity-enhancing β116Ala→Ser replacement that distinguishes high- and low-altitude variants. Replacement of the nonpolar Ala for an uncharged, polar Ser at the α_1_β_1_ intradimer contact surface is predicted to increase Hb-O_2_ affinity by stabilizing the R-state via intra-subunit hydrogen bonds between the γ-oxygen of β116Ser and each of three β-chain residues: β26Glu (ε2-oxygen), β113Val (carbonyl oxygen), and β117His (ε2-nitrogen). Homology modelling indicates that the same network of interhelical bonds is not present in the T-state.

The β116Ala→Ser substitution involves the replacement of nonpolar alanine for an uncharged, polar serine at an α_1_β_1_ intradimer contact. This replacement appears to increase O_2_-affinity by stabilizing the R-state via intra-subunit hydrogen bonds between the γ-oxygen of β116Ser and each of three β-chain residues: β26Glu (ε2-oxygen), β113Val (carbonyl oxygen), and β117His (ε2-nitrogen)(Fig 6). Consistent with this interpretation, mutations in human Hb that disrupt this same network of hydrogen bonds also exhibit increased O_2_-affinities [57]. This appears to represent a rare case in which Hb-O_2_ affinity is increased via stabilization of the R-state rather than destabilization of the T-state.

### Population Genomic Tests of Spatially Varying Selection

**Fig 7:**
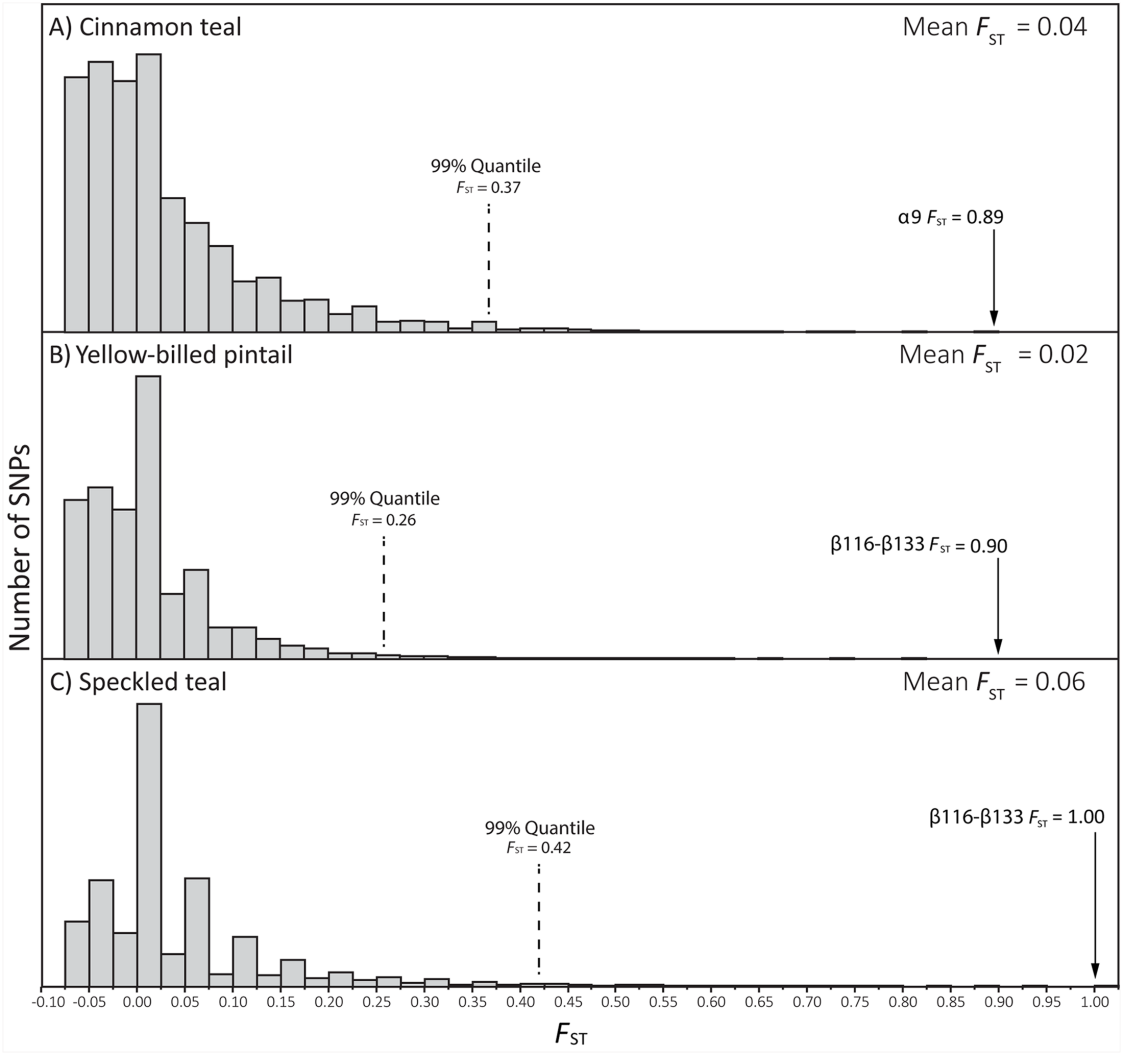
Function-altering amino acid polymorphisms in the α^*A*^- and β^*A*^-globin genes emerge as extreme outliers in genome scans of allele-frequency differentiation (*F*_ST_) between high- and low-altitude populations of speckled teal, cinnamon teal, and yellow-billed pintails. Histograms depict genome-wide distributions of SNP-specific *F*_ST_ values based on RAD-Seq surveys of genomic polymorphism in high- and low-altitude populations of (*A*) cinnamon teal (*n* = 18,145 SNPs), (*B*) yellow-billed pintails (*n* = 49,670 SNPs), and (*C*) speckled teal (*n* = 47,731 SNPs).

The fact that high-altitude taxa exhibited higher Hb-O_2_ affinities than their lowland sister taxa in six of eight pairwise comparisons is an intriguing trend and is suggestive of adaptive convergence, but the overall pattern does not permit conclusive inferences about the adaptive significance of observed changes in Hb function in any particular high-altitude population or species. In principle, genome-wide analyses of nucleotide differentiation between individual pairs of high- and low-altitude populations can provide an independent means of assessing whether altitudinal differences in globin allele frequencies may be attributable to a history of spatially varying selection. Accordingly, we used restriction-site associated DNA sequencing (RAD-Seq) to survey genome-wide patterns of nucleotide differentiation between high- and low-altitude populations of three separate species: cinnamon teal, yellow-billed pintail, and speckled teal. In each of these three pairwise population comparisons, function-altering amino acid polymorphisms in the α^*A*^- and/or β^*A*^-globin genes emerged as highly significant outliers in the genome-wide distribution of site-specific *F*_ST_ values (Fig 7). Indirect inferences about the adaptive significance of these polymorphisms are corroborated by results of the functional experiments, which demonstrated that the derived variants at these sites contributed to increases in Hb-O_2_ affinity in high-altitude populations of all three species (α^*A*^9 in cinnamon teal and the two-site ‘116–133’ β^*A*^-globin haplotypes shared by yellow-billed pintail and speckled teal). Since the β^*A*^-globin allele of high-altitude yellow-billed pintail was derived via introgressive hybridization with high-altitude speckled teal, the combined results of our functional experiments and population genomic analyses provide strong evidence for positive selection on introgressed allelic variants. This finding contributes to a growing body of evidence that introgressive hybridization can provide an important source of adaptive genetic variation in animal populations [58–60].

### Conclusions

Convergent increases in Hb-O_2_ affinity in high-altitude waterfowl taxa were caused by a combination of unique amino acid replacements (as in the case of cinnamon teal, where the causative mutation was not shared with other highland taxa), parallel replacements (as in the case of high-altitude crested ducks and Puna teal that shared independently derived β94Asp→Glu mutations), and collateral replacements (as in the case of yellow-billed pintail and speckled teal that shared identical-by-descent β-globin alleles due to a history of introgressive hybridization). Andean goose appears to represent another case where the evolution of a derived increase in Hb-O_2_ affinity is attributable to one or more unique substitutions, although additional experiments will be required to pinpoint the causative change(s). These results demonstrate that convergent changes in protein function can occur through multiple historical paths involving multiple possible mutations. Among the Andean waterfowl taxa that we examined, we identified only a single case where a convergent increase in Hb-O_2_ affinity was attributable to a true parallel amino acid substitution (β94Asp→Glu in high-altitude crested ducks and Puna teal).

The limited number of function-altering parallel substitutions in the Hbs of Andean waterfowl stands in contrast to patterns of functional evolution in vertebrate opsin proteins, where convergent changes in the wavelengths of maximum absorbance (spectral tuning) are very often attributable to parallel amino acid substitutions [61,62]. In vertebrate opsins, the more pervasive patterns of parallelism may reflect the fact that genetically based changes in spectral tuning can only be achieved via specific mutational replacements at a limited number of key residues in the active site [63]. Our findings are more consistent with results of experimental evolution studies in microbes and yeast where replicated changes in fitness involved little to no parallelism at the underlying sequence level [64,65].

Our comparative survey also identified numerous parallel substitutions that had no effect on the inherent oxygenation properties of Hb, although we cannot rule out the possibility that the derived variants contributed to changes in other structural or functional properties. Our results for waterfowl Hbs provide two important lessons about repeated evolution at the molecular level: (*i*) most cases of convergence in protein function did not involve true parallel substitutions (indicating that similar phenotypic outcomes can be produced by multiple possible mutations), and (*ii*) most parallel substitutions produced no change in Hb-O_2_ affinity (convergent or otherwise). These findings demonstrate that parallel substitutions cannot be interpreted as *prima facie* evidence for adaptive evolution [66,67], and that the functional significance (and, hence, adaptive significance) of specific substitutions needs to be experimentally tested in order to support conclusions about the molecular basis of phenotypic evolution.

## Materials and Methods

### Specimen Collection

Blood and tissue samples were obtained from Andean waterfowl at high- and low-altitude localities as described previously [35]. Samples from Orinoco geese, Abyssinian blue-winged geese, and Hartlaub’s ducks were obtained from Sylvan Heights Waterfowl Park (Scotland Neck, North Carolina). Animals were handled in accordance with protocols approved by the Institutional Animal Care and Use Committee of the University of Alaska (certification numbers 02-01-152985 and 05-05-152985).

### Characterization of Hb Isoform Composition

We characterized Hb isoform composition in the mature erythrocytes of 106 wild-caught birds (median sample size = 14 individuals per species) (S1 Table). Native Hb components were separated by means of IEF using precast Phast gels (pH 3–9) (GE Healthcare; 17-0543-01). IEF gel bands were excised and digested with trypsin, and MS/MS was used to identify the resultant peptides, as described previously [26,28,32,68]. Database searches of the resultant MS/MS spectra were performed using Mascot (Matrix Science, v1.9.0, London, UK); peptide mass fingerprints were queried against a custom database of avian globin sequences, including the full complement of embryonic and adult α- and β-type globin genes that have been annotated in avian genome assemblies [38,69–73]. We identified all significant protein hits that matched more than one peptide with *P*<0.05. After separating the HbA and HbD isoforms by native gel IEF, the relative abundance of the two isoforms was quantified densitometrically using Image J [74].

### Molecular Cloning and Sequencing

The α^*A*^- and β^*A*^-globin genes were amplified and sequenced according to protocols described previously [35,36]. For all specimens used as subjects in the experimental analyses of Hb function, we extracted RNA from whole blood using the RNeasy kit (Qiagen,Valencia, CA), and we amplified full-length cDNAs of the α^*D*^-globin gene using a OneStep RT-PCR kit (Qiagen, Valencia, CA). We designed paralog-specific primers using 5’ and 3’ UTR sequences, as described by Opazo et al. [38]. We cloned reverse transcription (RT)-PCR products into pCR4-TOPO vector using the TOPO TA Cloning Kit (Invitrogen, Carlsbad, CA), and we sequenced at least five clones per sample in order to recover both alleles. This enabled us to determine full diploid genotypes for α^*D*^-globin in each specimen. The sequences were analyzed using Geneious Pro ver. 5.4.3. All new sequences were deposited in GenBank under accessions numbers KT988975-KT988992 and KU160516-KU160529.

### Inferences of Character Polarity

For each amino acid difference between pairs of high- and low-altitude sister taxa, we identified ancestral and derived states by comparison with orthologous sites in a large number of other waterfowl species (*n* = 117 sequences for α^*A*^-globin, 96 for β^*A*^-globin, and 57 for α^*D*^-globin). Alignments of variable sites in the α^*A*^-, β^*A*^-, and α^*D*^-globin genes are shown in S3, S4 and S5 Figs, respectively. For each divergent site in each pair of sister taxa, unordered parsimony (using the trace character function in Mesquite [75]) yielded unambiguous inferences of character polarity.

### Inferring Causes of Molecular Homoplasy

One notable case of homoplasy in the β^*A*^-globin gene involved sites 116 and 133 in high-altitude yellow-billed pintails and speckled teal (S4 Fig), two species that are known to hybridize in nature [40,41]. To assess whether identical two-site ‘β116Ser-β133Met’ haplotypes from the two species were identical-by-descent, we reconstructed haplotype networks of β^*A*^-globin coding sequence using the median-joining algorithm [76], as implemented in the program Network 4.6 (Fluxus Technology, Suffolk, UK). We conducted the analysis on a sample of 257 β^*A*^-globin sequences (116 from yellow-billed pintails and 141 from speckled teal) obtained from sympatric high- and low-altitude populations of both species.

### RAD-Seq Analysis of Genome-Wide Nucleotide Differentiation

Sixty individuals representing three species of Andean ducks (speckled teal, cinnamon teal, and yellow-billed pintail) were selected for genome-wide surveys of nucleotide variation using single-digest RAD-Seq [77]. For each species, ten male specimens were selected from high-altitude (≥3,211 m above sea level), and ten were selected from low-altitude (≤ 914 m). Total genomic DNA was extracted from muscle tissue using a DNeasy Tissue Kit (Qiagen, Valencia, California, USA) and normalized using a Qubit Fluorometer (Invitrogen, Grand Island, New York, USA). DNA samples were submitted to Floragenex (Eugene, Oregon, USA) for single-digest RAD-Seq using *SbfI*, which recognizes an 8-nucleotide (CCTGCAGG) restriction site. Digested DNAs were ligated to barcodes and sequencing adaptors and then sequenced on the Illumina HiSeq 2000 with single-end 100 bp chemistry. Following Illumina sequencing, sequences were demultiplexed and trimmed to yield RAD sequences of 90 bp. Data analysis and bioinformatics pipelines were provided by Floragenex [77–79].

The Floragenex RAD unitag assembler and BSP pipelines v.2.0 were used to create a RAD-Seq ‘unitag’ assembly and Bowtie alignments of SAMtools pileup sequences to the reference assembly. Genotypes at each nucleotide site were determined using the VCF popgen v.4.0 pipeline to generate a customized VCF 4.1 (variant call format) database with parameters set as follows: minimum AF for genotyping = 0.075, minimum Phred score = 15, minimum depth of sequencing coverage = 10x, and allowing missing genotypes from up to 10% of individuals at each site.

To filter out base calls that were not useful due to low quality scores or insufficient coverage, genotypes at each nucleotide site were inferred using the Bayesian maximum likelihood algorithm described by Hohenlohe et al. [79]. This algorithm calculates the likelihood of each possible genotype at each site using a multinomial sampling distribution, which gives the probability of observing a set of read counts (*n*_*1*_, *n*_*2*_, *n*_*3*_, *n*_*4*_) for a particular genotype, where *n*_*i*_ is the read count for each of the four possible nucleotides at each site, excluding ambiguous reads with low quality scores. The genotyping algorithm incorporates the site-specific sequencing error rate, and assigns the most likely diploid genotype to each site using a likelihood ratio test and significance level of α = 0.05.

A total of 372 million sequence reads were obtained with an average depth of 7.6 (±2.4 SD) million reads per sample for yellow-billed pintail and speckled teal and 3.3 (±1.4 SD) million reads per sample for cinnamon teal, corresponding to an average of 140,671 (±27,856) RAD loci. After filtering and genotyping, the RAD-Seq survey yielded 49,670 SNPs associated with 18,998 distinct loci in yellow-billed pintail, 47,731 SNPs associated with 19,433 distinct loci in speckled teal, and 18,145 SNPs associated with 9,300 distinct loci in cinnamon teal, respectively. The mean depth of coverage was 36.8 (±10.0 SD) reads per site with an average per site quality score of 166.2 (±31.3 SD) for yellow-billed pintail and speckled teal, and 39.8 (±24.4 SD) reads per site with an average per site quality score of 177.6 (±26.4 SD) for cinnamon teal. Illumina reads were submitted to the European Nucleotide Archive and can be accessed under the short read archive (SRA) accession number PRJEB11624.

Sequencing coverage and quality scores were summarized using the software VCFtools v.0.1.11 [80]. Custom perl scripts were first used to filter triploid or tetraploid sites and convert the Floragenex-generated VCF file to a biallelic, VCF v4.0 compatible format. We then calculated Weir and Cockerham’s [81] estimator of *F*_ST_ for each SNP in comparisons between high- and low-altitude population samples.

### Protein Purification and *In Vitro* Analysis of Hb Function

We purified HbA and HbD variants from hemolysates of 1–4 specimens per species, all of which had known α^*A*^-, α^*D*^-, and β^*A*^-globin genotypes. In the case of ruddy ducks and yellow-billed pintails, previous population surveys of sequence polymorphism in the α^*A*^- and β^*A*^-globin genes had revealed multiple amino acid haplotypes segregating within high- and/or low-altitude populations [35,36]. In each case we purified HbA and HbD variants from individuals that were homozygous for each of the alternative allelic variants. Hemolysates of each individual specimen were dialyzed overnight against 20 mM Tris buffer (pH 8.4). The two tetrameric HbA and HbD isoforms were then separated using a HiTrap Q-HP column (GE Healthcare; 1 ml 17-1153-01) and equilibrated with 20 mM Tris buffer (pH 8.4). HbD was eluted against a linear gradient of 0–200 mM NaCl. The samples were desalted by means of dialysis against 10 mM HEPES buffer (pH 7.4) at 4°C, and were then concentrated by using a 30 kDa centrifuge filter (Amicon, EMD Millipore).

We measured O_2_-equilibria of purified Hb solutions under standard conditions (37°C, pH 7.4, 0.3 mM heme) using a modified diffusion chamber where absorption at 436 nm was monitored during stepwise changes in equilibration gas mixtures generated by precision Wösthoff gas-mixing pumps [28,32,39,56,82,83]. In order to characterize intrinsic Hb-O_2_ affinities and mechanisms of allosteric regulatory control, we measured O_2_-equilibria in the presence of Cl^-^ ions (0.1M KCl), in the presence of IHP (IHP/Hb tetramer ratio = 2.0), in the simultaneous presence of both effectors, and in the absence of both effectors (stripped). Free Cl^-^ concentrations were measured with a model 926S Mark II chloride analyzer (Sherwood Scientific Ltd, Cambridge, UK). We estimated values of *P*_50_ and *n*_50_ (Hill’s cooperativity coefficient at half-saturation) by fitting the Hill equation *Y* = *P*O_2_^*n*^/(*P*_50_^*n*^ + *P*O_2_^*n*^) to the experimental O_2_ saturation data by means of nonlinear regression (*Y* = fractional O_2_ saturation; *n*, cooperativity coefficient). The model-fitting was based on 5–8 equilibration steps between 30% and 70% oxygenation.

### Vector Construction and Site-Directed Mutagenesis

The α^*A*^- and β^*A*^-globin sequences of yellow-billed pintail were synthesized by Eurofins MWG Operon (Huntsville, AL, USA) after optimizing the nucleotide sequences in accordance with *E*. *coli* codon preferences. The synthesized α^*A*^-β^*A*^ globin gene cassette was cloned into a custom pGM vector system along with the *methionine aminopeptidase* (MAP) gene, as described by Natarajan et al. [27,84]. We engineered each of the β-chain codon substitutions using the QuikChange II XL Site-Directed Mutagenesis kit from Stratagene (LaJolla, CA, USA). Each engineered codon change was verified by DNA sequencing.

### Expression and Purification of Recombinant Hbs

Recombinant Hb expression was carried out in the JM109 (DE3) *E*. *coli* strain as described in Natarajan et al. [27,84]. To ensure the post-translational cleaving of N-terminal methionines from the nascent globin chains, we co-transformed a plasmid (pCO-MAP) containing an additional copy of the *MAP* gene. Both pGM and pCO-MAP plasmids were cotransformed and subject to dual selection in an LB agar plate containing ampicillin and kanamycin. The expression of each rHb mutant was carried out in 1.5 L of TB medium. Bacterial cells were grown in 37°C in an orbital shaker at 200 rpm until absorbance values reached 0.6–0.8 at 600 nm. The bacterial cultures were induced by 0.2 mM IPTG and were then supplemented with hemin (50 μg/ml) and glucose (20 g/L). The bacterial culture conditions and the protocol for preparing cell lysates were described previously [27–29,32,84].

The bacterial cells were resuspended in lysis buffer (50 mM Tris, 1 mM EDTA, 0.5 mM DTT, pH 7.6) with lysozyme (1 mg/g wet cells) and were incubated in the ice bath for 30 min. Following sonication of the cells, 0.5–1.0% polyethylenimine solution was added, and the crude lysate was then centrifuged at 15000 g for 45 min at 4°C. The rHbs were purified by two-step ion-exchange chromatography. Using high-performance liquid chromatography, the samples were passed through a prepacked anion-exchange column (Q-Sepharose) followed by passage through a cation-exchange column (SP-Sepharose). The clarified supernatant was subjected to overnight dialysis in CAPS buffer (20 mM CAPS with 0.5mM EDTA, pH 9.7) at 4°C. The samples were passed through the Q-column and the rHb solutions were eluted against a linear gradient of 0–1.0 M NaCl. The eluted samples were desalted by overnight dialysis with 20 mM HEPES pH 7.4 (4°C). Dialyzed samples were then passed through the SP-Sepharose column (HiTrap SPHP, 1 mL, 17-1151-01; GE Healthcare) equilibrated with 20 mM HEPES (pH 7.4). The rHb samples were eluted with a linear gradient of 20 mM HEPES (pH 9.2). Samples were concentrated and desalted by overnight dialysis against 10 mM HEPES buffer (pH 7.4) and were stored at -80°C prior to the measurement of O_2_-equilibrium curves.

The purified rHb samples were analyzed by means of sodium dodecyl sulphate (SDS)-polyacrylamide gel electrophoresis. After preparing rHb samples as oxyHb, deoxyHb, and carbonmonoxy derivatives, we measured absorbance at 450–600 nm to confirm that the absorbance maxima match those of the native HbA samples. Results of isoelectric focusing analyses indicated that each of the purified rHb mutants was present as a tetrameric assembly, and this was further confirmed by cooperativity coefficients (*n*_50_) >1.00 in the O_2_-equilibrium experiments. *In vitro* measurements of O_2_-binding properties were conducted in the same manner for rHbs and native Hb samples.

### Structural Modeling

Homology-based structural modeling was performed with Modeller 9.15 [85] using human Hbs in different ligation states (PDB, 2hhb and 1hho) as templates. Models were evaluated on the SWISS-MODEL server [86]. All models had QMEAN values between 0.71 and 0.78. Structural mining was performed using PISA [87], PyMol (Schrödinger, New York, NY), and SPACE [88].

## Supporting Information

S1 Fig Diagram illustrating the allosteric regulation of Hb-O_2_ affinity. (*A*) The oxygenation reaction of tetrameric Hb (α_2_β_2_) involves an allosteric transition in quaternary structure from the low-affinity T-state to the high-affinity R-state. The oxygenation-induced T→R transition entails a breakage of salt bridges and hydrogen bonds within and between subunits (open squares), dissociation of allosterically bound organic phosphates (OPHs), Cl^-^ ions, and protons, and the release of heat (heme oxygenation is an exothermic reaction). Deoxygenation-linked proton binding occurs at multiple residues in the α- and β-chains, Cl^-^ binding mainly occurs at the N-terminal α-amino groups of the α- and β-chains in addition to other residues in both chains, and phosphate binding occurs between the β-chains in the central cavity of the Hb tetramer. (*B*) O_2_-equilibrium curves for purified Hb in the absence of allosteric effectors (stripped) and in the presence of chloride ions (+Cl^-^) and organic phosphates (+OPH). The preferential binding of allosteric effectors to deoxyHb stabilizes the T-state, thereby shifting the allosteric equilibrium in favor of the low-affinity quaternary structure. The O_2_-equilibrium curves are therefore right-shifted (Hb-O_2_ affinity is reduced) in the presence of allosteric effectors. Hb-O_2_ affinity is indexed by the *P*_50_ value—the *P*O_2_ at which Hb is half-saturated. The sigmoidal shape of the O_2_-equilibrium curves reflects cooperative O_2_-binding, involving a *P*O_2_-dependent shift from low- to high-affinity conformations.
(PDF)

S2 Fig Alignment of α^*A*^-, α^*D*^-, and β^*A*^-globin amino acid sequences in the set of focal waterfowl taxa. (PDF)

S3 Fig Alignment of amino acid sites in the α^*A*^-globin gene that differ in one or more comparisons between high- and low-altitude sister taxa (highlighted in yellow). Character states at orthologous sites in a phylogenetically diverse set of waterfowl taxa (*n* = 117 orthologous sequences) permitted unambiguous inferences regarding the polarity of observed amino acid substitutions.
(PDF)

S4 Fig Alignment of amino acid sites in the β^*A*^-globin gene that differ in one or more comparisons between high- and low-altitude sister taxa (highlighted in yellow). Character states at orthologous sites in a phylogenetically diverse set of waterfowl taxa (*n* = 96 orthologous sequences) permitted unambiguous inferences regarding the polarity of observed amino acid substitutions.
(PDF)

S5 Fig Alignment of amino acid sites in the α^*D*^-globin gene that differ in one or more comparisons between high- and low-altitude sister taxa (highlighted in yellow). Character states at orthologous sites in a phylogenetically diverse set of waterfowl taxa (*n* = 57 orthologous sequences) permitted unambiguous inferences regarding the polarity of observed amino acid substitutions.
(PDF)

S6 Fig Network of β^*A*^-globin haplotypes sampled from sympatric highland and lowland populations of yellow-billed pintail and speckled teal. The median-joining network reconstruction was based on a total of 257 DNA sequence haplotypes (*n* = 116 and 141 β^*A*^-globin sequences for yellow-billed pintail and speckled teal, respectively). The sharing of ‘β116Ser-β133Met’ alleles between highland populations of both species reflects a history of introgressive hybridization.
(PDF)

S7 Fig Oxygenation properties of HbA and HbD isoforms from high- and low-altitude populations of cinnamon teal, *Anas cyanoptera*. (A) *P*_50_ values (means ± SEM) for purified HbA variants of highland and lowland teal measured at pH 7.4 and 37°C in the absence (stripped) and presence of allosteric effectors ([Cl^-^], 0.1 M; [HEPES], 0.1 M; IHP/Hb tetramer ratio, 2.0; [Heme], 0.300 mM). (B) Log-transformed differences in *P*_50_ values of highland and lowland HbA variants in the presence and absence of allosteric effectors. The Δlog-*P*_50_ values measure the extent to which Hb-O_2_ affinity is reduced in the presence of a given allosteric effector (Cl^-^, IHP, or both anions together). (C) *P*_50_ values for HbD variants of highland and lowland teal (experimental conditions as above). (D) Δlog-*P*_50_ values for HbD variants of highland and lowland teal.
(PDF)

S8 Fig Oxygenation properties of HbA and HbD isoforms from Andean goose (*Chloephaga melanoptera*), a high-altitude native, and Orinoco goose (*Neochen jubata*), a low-altitude native. (A) *P*_50_ values (means ± SEM) for purified HbA isoforms of the two species measured at pH 7.4 and 37°C in the absence (stripped) and presence of allosteric effectors ([Cl^-^], 0.1 M; [HEPES], 0.1 M; IHP/Hb tetramer ratio, 2.0; [Heme], 0.300 mM). (B) Log-transformed differences in *P*_50_ values of HbA isoforms of the two species in the presence and absence of allosteric effectors. The Δlog-*P*_50_ values measure the extent to which Hb-O_2_ affinity is reduced in the presence of a given allosteric effector (Cl^-^, IHP, or both anions together). (C) *P*_50_ values for HbD isoforms of Andean goose and Orinoco goose (experimental conditions as above). (D) Δlog-*P*_50_ values for HbD isoforms of the two species.
(PDF)

S9 Fig Oxygenation properties of HbA and HbD isoforms from high- and low-altitude populations of crested ducks, *Lophonetta specularioides*. (A) *P*_50_ values (means ± SEM) for purified HbA variants of highland and lowland ducks measured at pH 7.4 and 37°C in the absence (stripped) and presence of allosteric effectors ([Cl^-^], 0.1 M; [HEPES], 0.1 M; IHP/Hb tetramer ratio, 2.0; [Heme], 0.300 mM). (B) Log-transformed differences in *P*_50_ values of highland and lowland HbA variants in the presence and absence of allosteric effectors. The Δlog-*P*_50_ values measure the extent to which Hb-O_2_ affinity is reduced in the presence of a given allosteric effector (Cl^-^, IHP, or both anions together). (C) *P*_50_ values for HbD variants of highland and lowland ducks (experimental conditions as above). (D) Δlog-*P*_50_ values for HbD variants of highland and lowland ducks.
(PDF)

S10 Fig Oxygenation properties of HbA and HbD isoforms from Puna teal (*Anas puna*), a high-altitude native, and silver teal (*Anas versicolor*), a low-altitude native. (A) *P*_50_ values (means ± SEM) for purified HbA isoforms of the two species measured at pH 7.4 and 37°C in the absence (stripped) and presence of allosteric effectors ([Cl^-^], 0.1 M; [HEPES], 0.1 M; IHP/Hb tetramer ratio, 2.0; [Heme], 0.300 mM). (B) Log-transformed differences in *P*_50_ values of HbA isoforms of the two species in the presence and absence of allosteric effectors. The Δlog-*P*_50_ values measure the extent to which Hb-O_2_ affinity is reduced in the presence of a given allosteric effector (Cl^-^, IHP, or both anions together). (C) *P*_50_ values for HbD isoforms of Puna teal and silver teal (experimental conditions as above). (D) Δlog-*P*_50_ values for HbD isoforms of the two species.
(PDF)

S11 Fig Oxygenation properties of HbA and HbD isoforms from high- and low-altitude populations of yellow-billed pintail, *Anas georgica*, and HbA isoforms from a pair of high- and low-altitude African species, Abyssinian blue-winged goose (*Cyanochen cyanoptera*) and Hartlaub’s duck (*Pteronetta hartlaubii*), that share one of the same β-chain substitutions. (A) *P*_50_ values (means ± SEM) for purified HbA variants of highland and lowland pintails measured at pH 7.4 and 37°C in the absence (stripped) and presence of allosteric effectors ([Cl^-^], 0.1 M; [HEPES], 0.1 M; IHP/Hb tetramer ratio, 2.0; [Heme], 0.300 mM). (B) Log-transformed differences in *P*_50_ values of highland and lowland HbA variants in the presence and absence of allosteric effectors. (C) *P*_50_ values for HbD variants of highland and lowland pintails (experimental conditions as above). (D) Δlog-*P*_50_ values for HbD variants of highland and lowland pintails. (E) *P*_50_ values (means ± SEM) for purified HbA isoforms of the blue-winged goose and Hartlaub’s duck (experimental conditions as above). (F) Δlog-*P*_50_ values for HbA isoforms of the blue-winged goose and Hartlaub’s duck.
(PDF)

S1 Table Percentage concentrations of the HbA and HbD isoforms (mean ± SD [same for both values]) in the red blood cells of high- and low-altitude waterfowl taxa. All data are from wild-caught specimens. Sample sizes (no. individuals) are given in parentheses.
(DOCX)

S2 Table O_2_ affinities (*P*_50_, torr) and cooperativity coefficients (*n*_50_) of purified HbA and HbD isoforms from highland and lowland waterfowl. O_2_ equilibria were measured in 0.1 mM HEPES buffer at pH 7.4 (± 0.01) and 37°C in the absence (stripped) and presence of Cl^-^ ions ([KCl]) and IHP (at two-fold molar excess over tetrameric Hb). *P*_50_ and *n*_50_ values were derived from single O_2_ equilibrium curves, where each value was interpolated from linear Hill plots (correlation coefficient *r*> 0.995) based on 4 or more equilibrium steps between 25 and 75% saturation. Due to allelic polymorphism, two alternative Hb variants were present in the low-altitude sample of ruddy ducks (‘low1’ and ‘low2’) and in the high-altitude sample of speckled teal (‘high1’ and ‘high2’). In the case of the ruddy ducks, ‘low 1’ and ‘low 2’ represent triply homozygous β^*A*^-globin genotypes ‘13Ser-14Ile-69Ser’ and ‘13Gly-14Leu-69Thr’, respectively. In the case of speckled teal, ‘high 1’ and ‘high 2’ represent triply homozygous β^*A*^-globin genotypes ‘13Ser-116Ser-133Met’ and ‘13Gly-116Ser-133Met’, respectively.
(DOCX)
