# Supplementary figures and images for: Convergent Evolution of Hemoglobin Function in High-Altitude Andean Waterfowl Involves Limited Parallelism at the Molecular Sequence Level
Source: PLoS Genet. 2015 Dec 4;11(12):e1005681. doi: 10.1371/journal.pgen.1005681 (PMC4670201; doi:10.1371/journal.pgen.1005681)

A

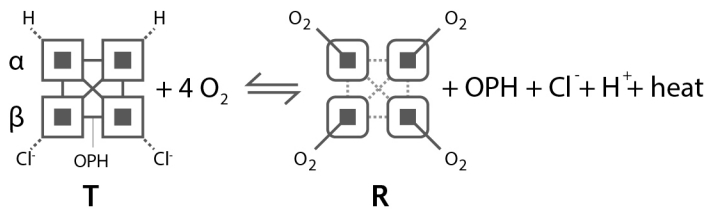

B

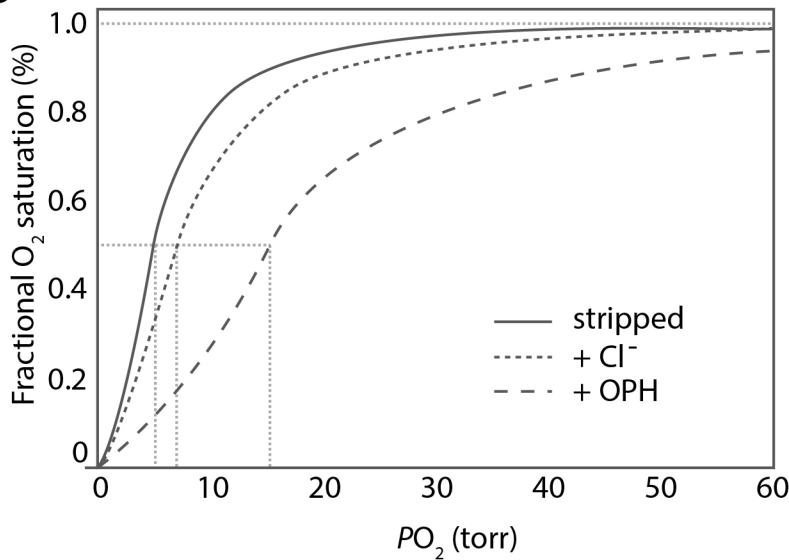

Supplement: S1 Fig — (A) The oxygenation reaction of tetrameric Hb (α2β2) involves an allosteric transition in quaternary structure from the low-affinity T-state to the high-affinity R-state. The oxygenation-induced T→R transition entails a breakage of salt bridges and hydrogen bonds within and between subunits (open squares), dissociation of allosterically bound organic phosphates (OPHs), Cl- ions, and protons, and the release of heat (heme oxygenation is an exothermic reaction). Deoxygenation-linked proton binding occurs at multiple residues in the α- and β-chains, Cl- binding mainly occurs at the N-terminal α-amino groups of the α- and β-chains in addition to other residues in both chains, and phosphate binding occurs between the β-chains in the central cavity of the Hb tetramer. (B) O2-equilibrium curves for purified Hb in the absence of allosteric effectors (stripped) and in the presence of chloride ions (+Cl-) and organic phosphates (+OPH). The preferential binding of allosteric effectors to deoxyHb stabilizes the T-state, thereby shifting the allosteric equilibrium in favor of the low-affinity quaternary structure. The O2-equilibrium curves are therefore right-shifted (Hb-O2 affinity is reduced) in the presence of allosteric effectors. Hb-O2 affinity is indexed by the P 50 value—the PO2 at which Hb is half-saturated. The sigmoidal shape of the O2-equilibrium curves reflects cooperative O2-binding, involving a PO2-dependent shift from low- to high-affinity conformations. (PDF) [file pgen.1005681.s001.pdf]

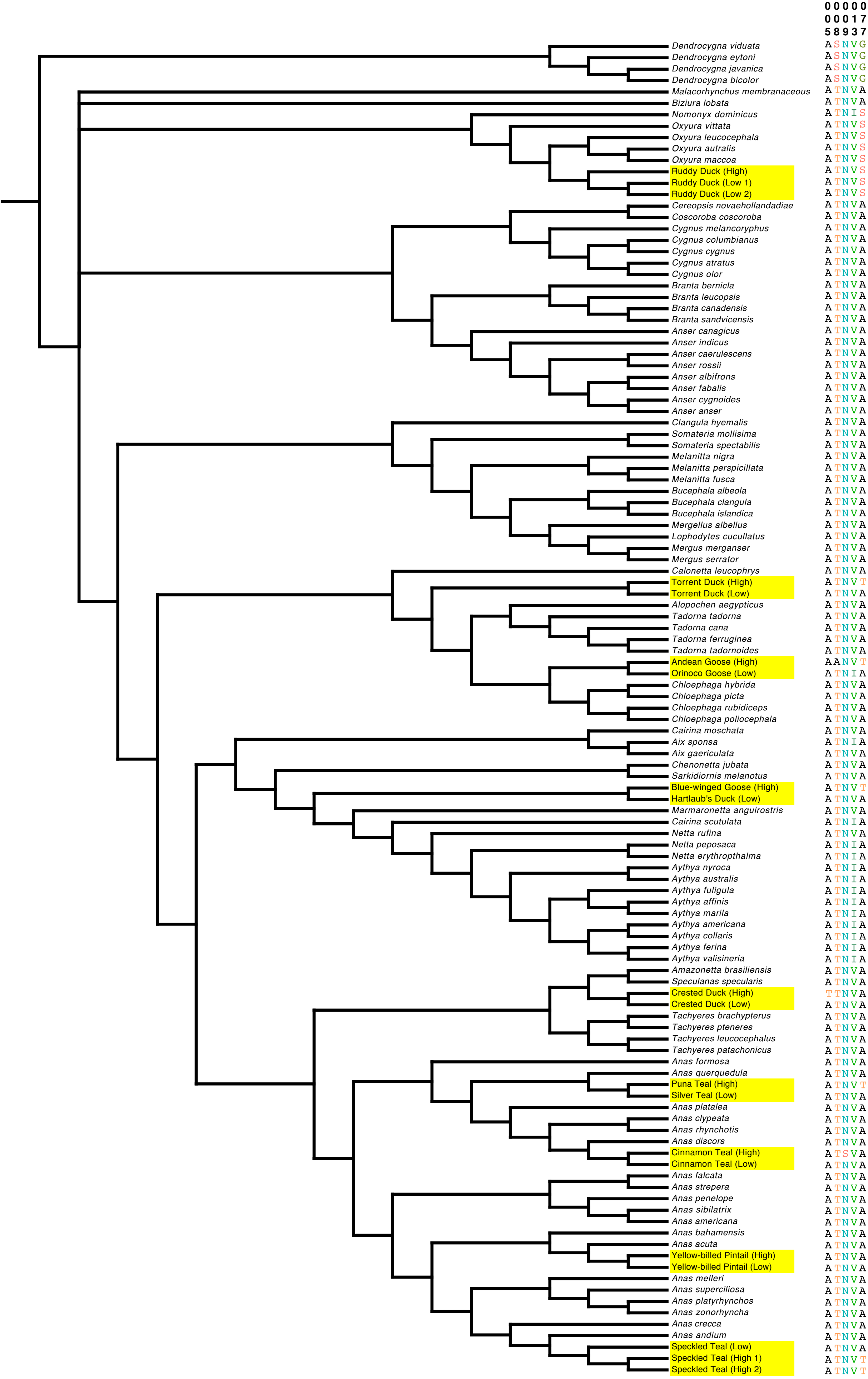

Supplement: S3 Fig — Character states at orthologous sites in a phylogenetically diverse set of waterfowl taxa (n = 117 orthologous sequences) permitted unambiguous inferences regarding the polarity of observed amino acid substitutions. (PDF) [file pgen.1005681.s003.pdf]

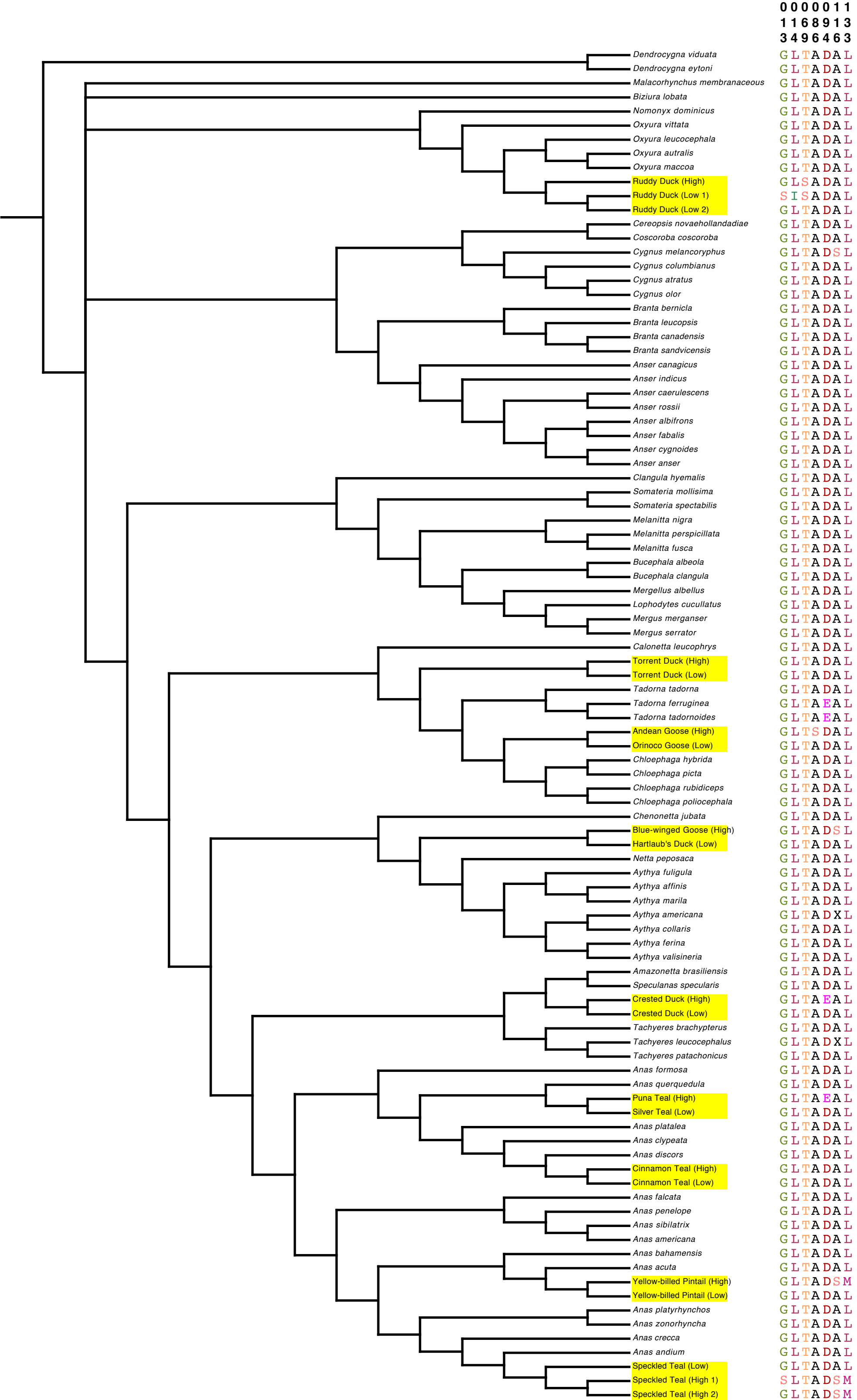

Supplement: S4 Fig — Character states at orthologous sites in a phylogenetically diverse set of waterfowl taxa (n = 96 orthologous sequences) permitted unambiguous inferences regarding the polarity of observed amino acid substitutions. (PDF) [file pgen.1005681.s004.pdf]

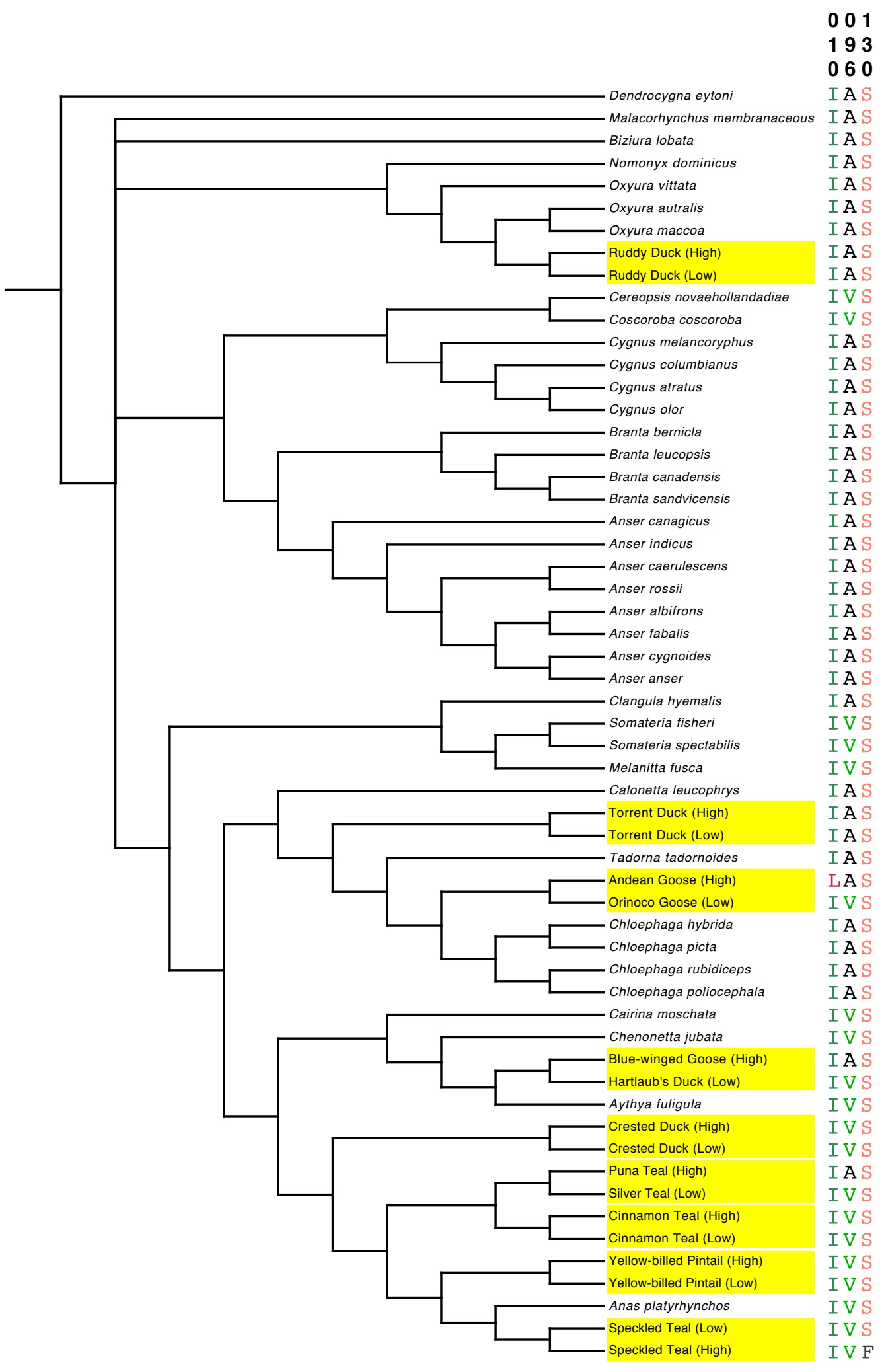

Supplement: S5 Fig — Character states at orthologous sites in a phylogenetically diverse set of waterfowl taxa (n = 57 orthologous sequences) permitted unambiguous inferences regarding the polarity of observed amino acid substitutions. (PDF) [file pgen.1005681.s005.pdf]

$\beta 116\text{Ser} + \beta 133\text{Met}$

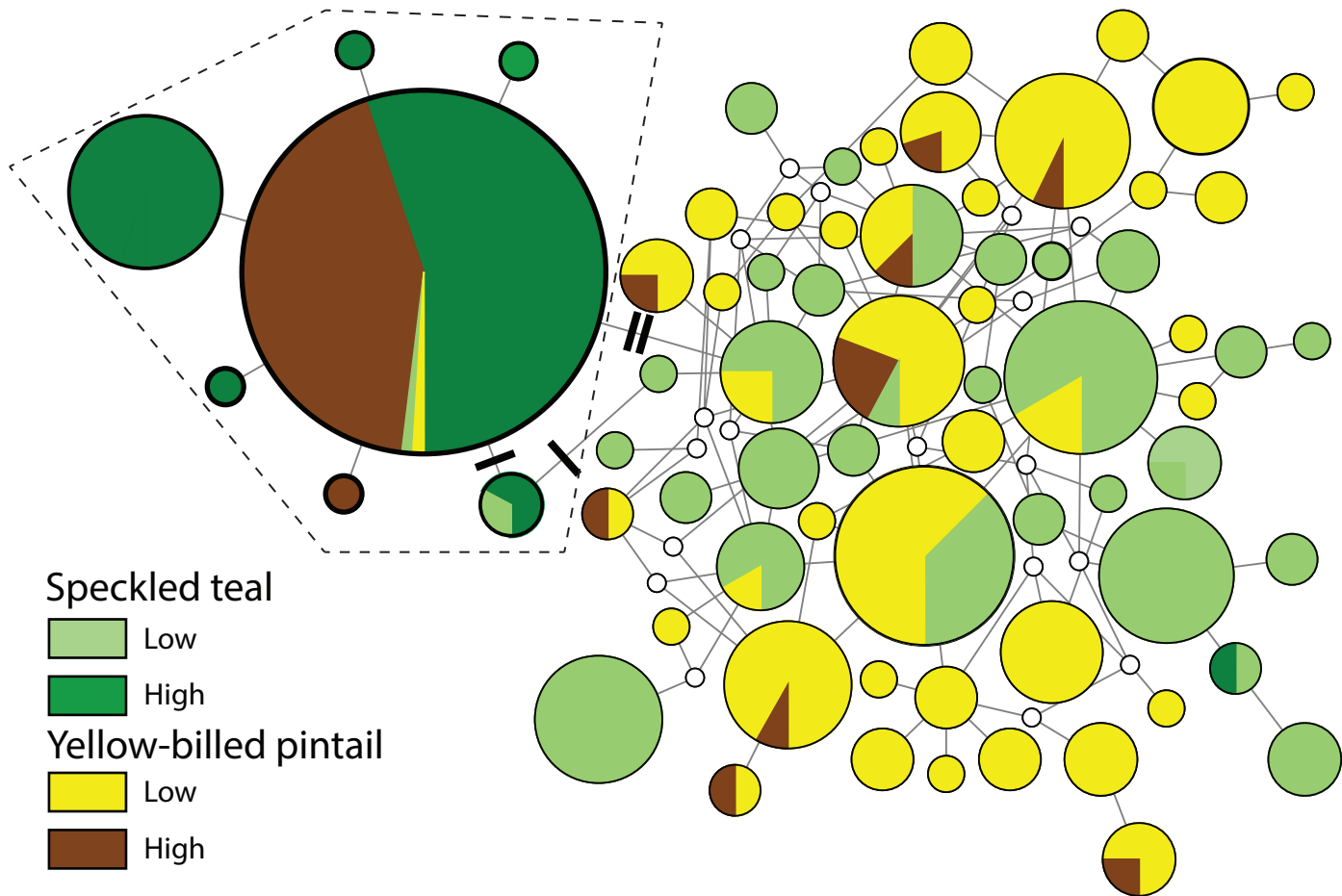

Supplement: S6 Fig — The median-joining network reconstruction was based on a total of 257 DNA sequence haplotypes (n = 116 and 141 βA-globin sequences for yellow-billed pintail and speckled teal, respectively). The sharing of ‘β116Ser-β133Met’ alleles between highland populations of both species reflects a history of introgressive hybridization. (PDF) [file pgen.1005681.s006.pdf]

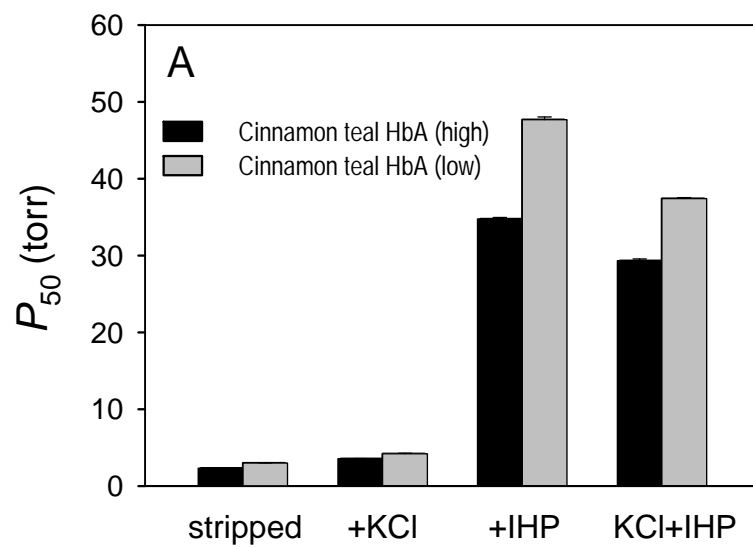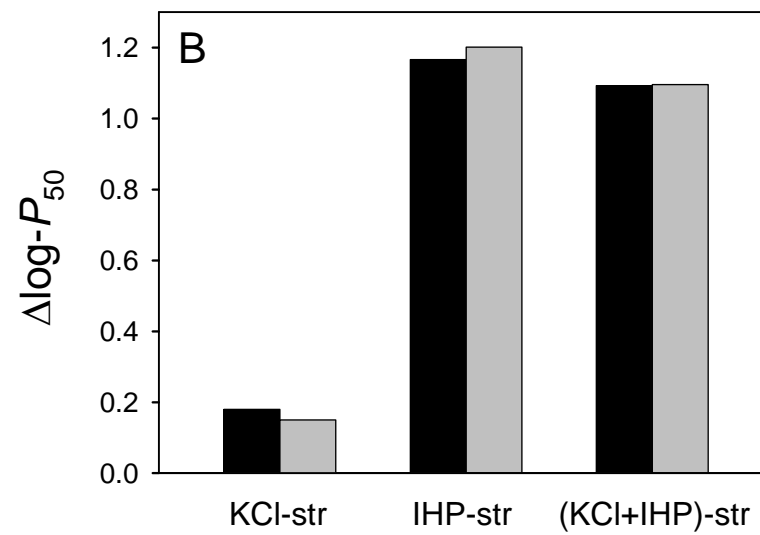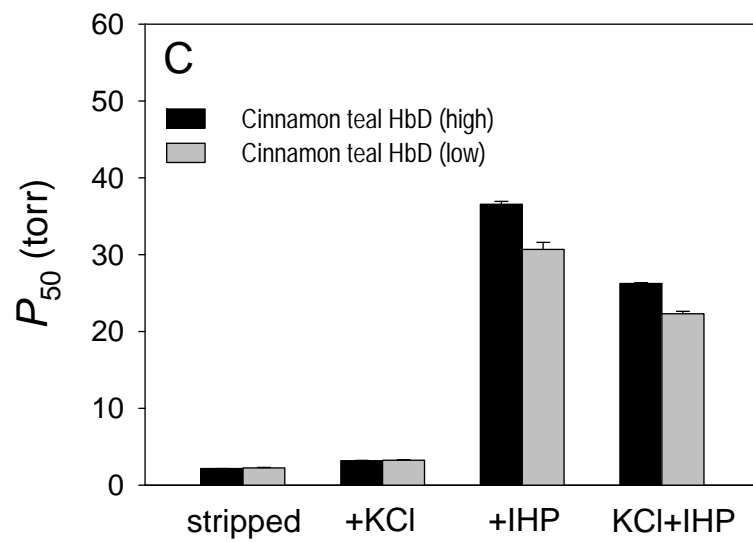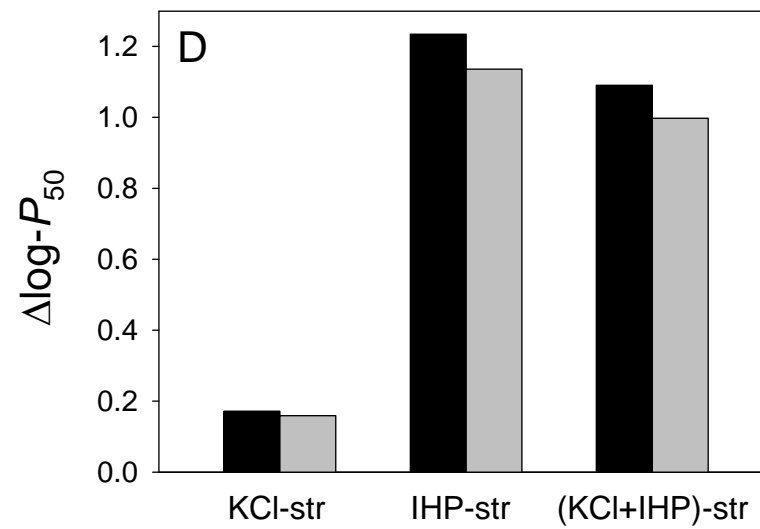

Supplement: S7 Fig — (A) P 50 values (means ± SEM) for purified HbA variants of highland and lowland teal measured at pH 7.4 and 37°C in the absence (stripped) and presence of allosteric effectors ([Cl-], 0.1 M; [HEPES], 0.1 M; IHP/Hb tetramer ratio, 2.0; [Heme], 0.300 mM). (B) Log-transformed differences in P 50 values of highland and lowland HbA variants in the presence and absence of allosteric effectors. The Δlog-P 50 values measure the extent to which Hb-O2 affinity is reduced in the presence of a given allosteric effector (Cl-, IHP, or both anions together). (C) P 50 values for HbD variants of highland and lowland teal (experimental conditions as above). (D) Δlog-P 50 values for HbD variants of highland and lowland teal. (PDF) [file pgen.1005681.s007.pdf]

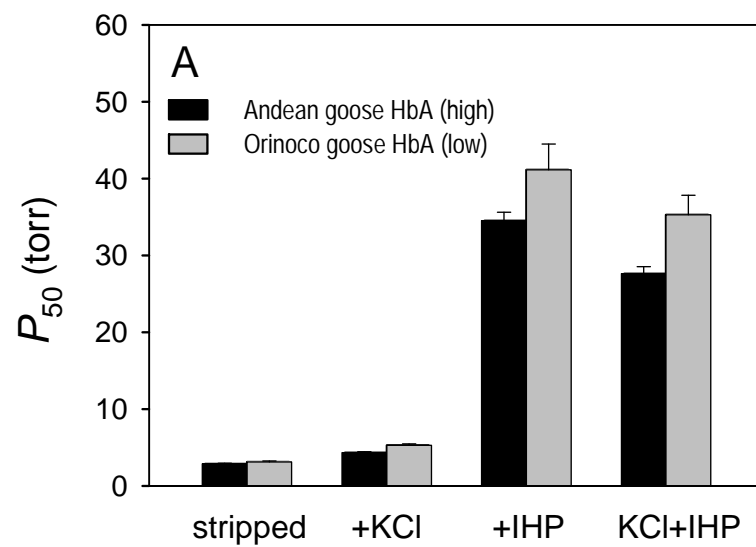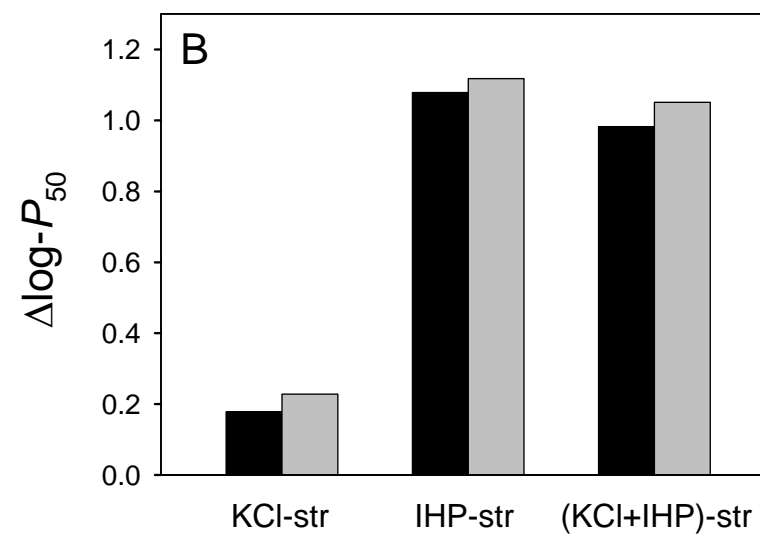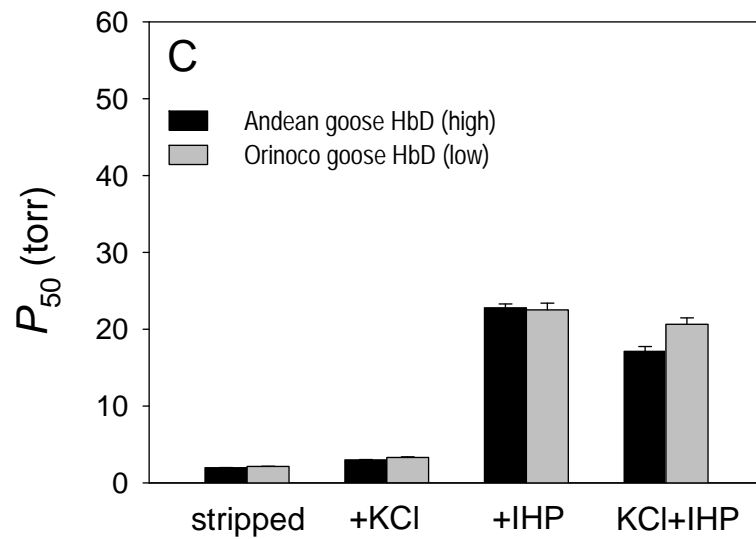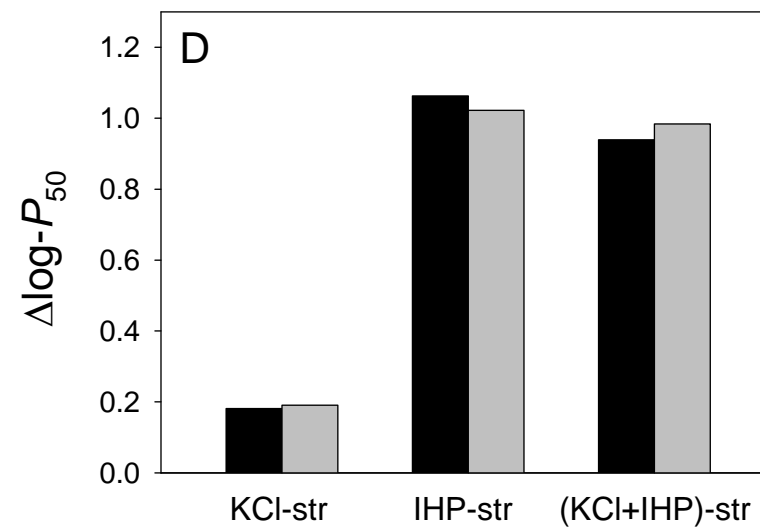

Supplement: S8 Fig — (A) P 50 values (means ± SEM) for purified HbA isoforms of the two species measured at pH 7.4 and 37°C in the absence (stripped) and presence of allosteric effectors ([Cl-], 0.1 M; [HEPES], 0.1 M; IHP/Hb tetramer ratio, 2.0; [Heme], 0.300 mM). (B) Log-transformed differences in P 50 values of HbA isoforms of the two species in the presence and absence of allosteric effectors. The Δlog-P 50 values measure the extent to which Hb-O2 affinity is reduced in the presence of a given allosteric effector (Cl-, IHP, or both anions together). (C) P 50 values for HbD isoforms of Andean goose and Orinoco goose (experimental conditions as above). (D) Δlog-P 50 values for HbD isoforms of the two species. (PDF) [file pgen.1005681.s008.pdf]

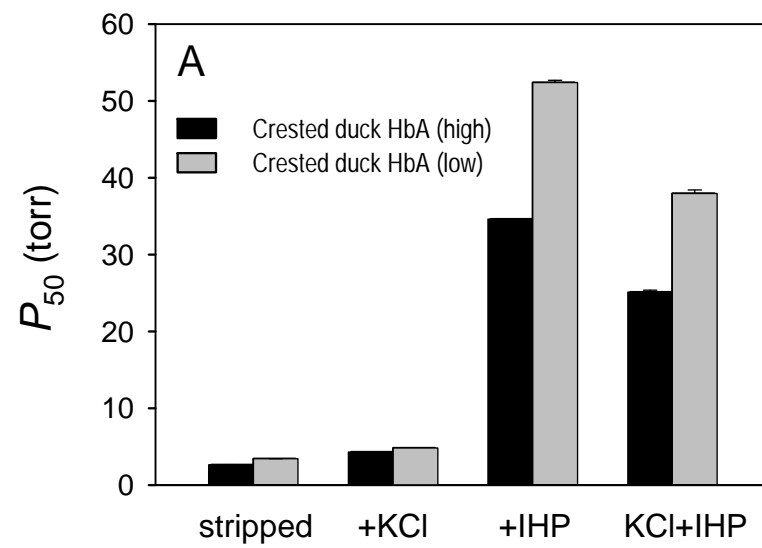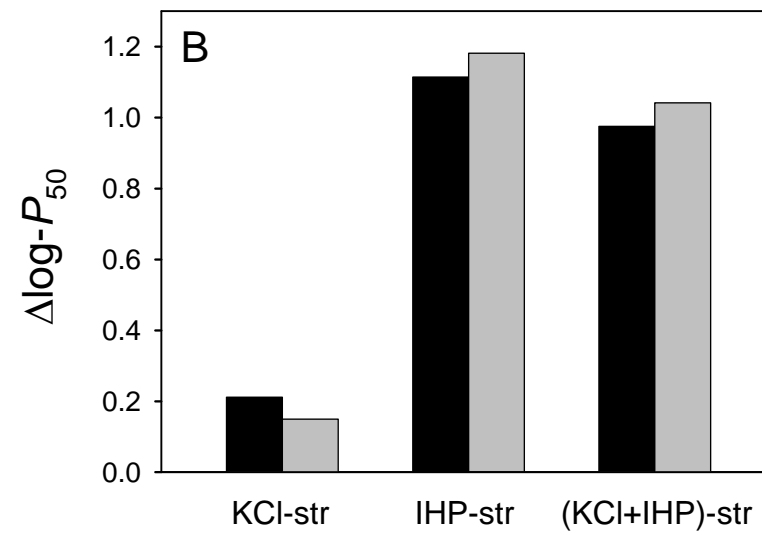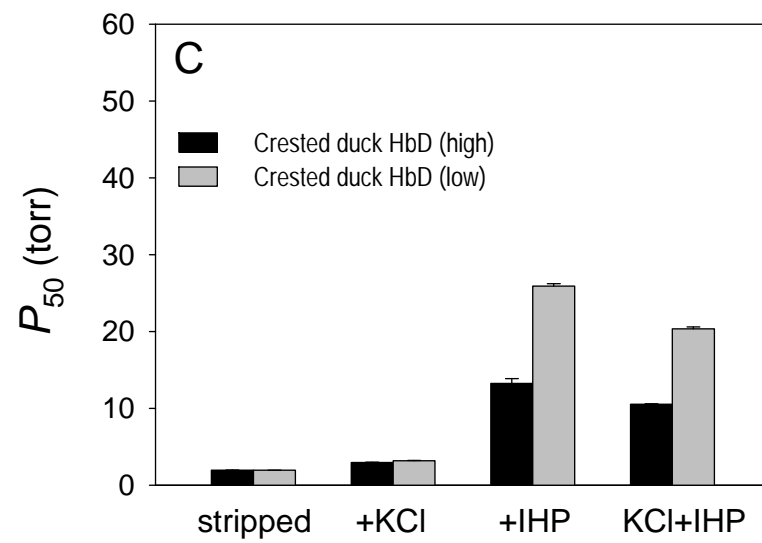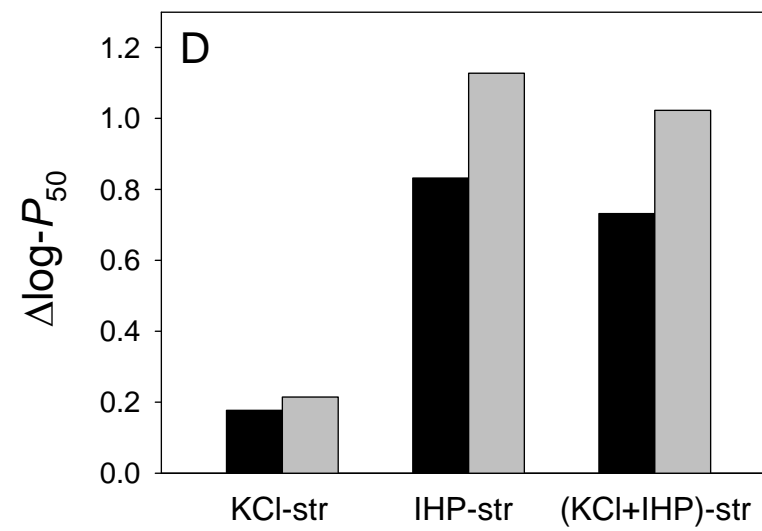

Supplement: S9 Fig — (A) P 50 values (means ± SEM) for purified HbA variants of highland and lowland ducks measured at pH 7.4 and 37°C in the absence (stripped) and presence of allosteric effectors ([Cl-], 0.1 M; [HEPES], 0.1 M; IHP/Hb tetramer ratio, 2.0; [Heme], 0.300 mM). (B) Log-transformed differences in P 50 values of highland and lowland HbA variants in the presence and absence of allosteric effectors. The Δlog-P 50 values measure the extent to which Hb-O2 affinity is reduced in the presence of a given allosteric effector (Cl-, IHP, or both anions together). (C) P 50 values for HbD variants of highland and lowland ducks (experimental conditions as above). (D) Δlog-P 50 values for HbD variants of highland and lowland ducks. (PDF) [file pgen.1005681.s009.pdf]

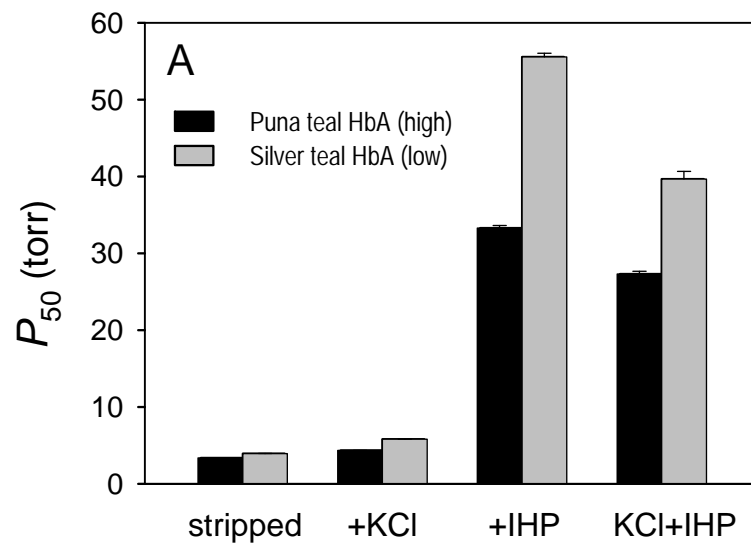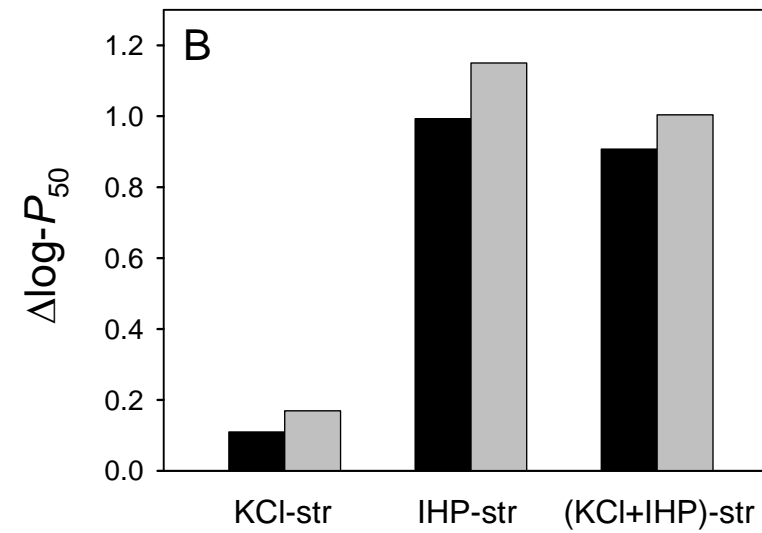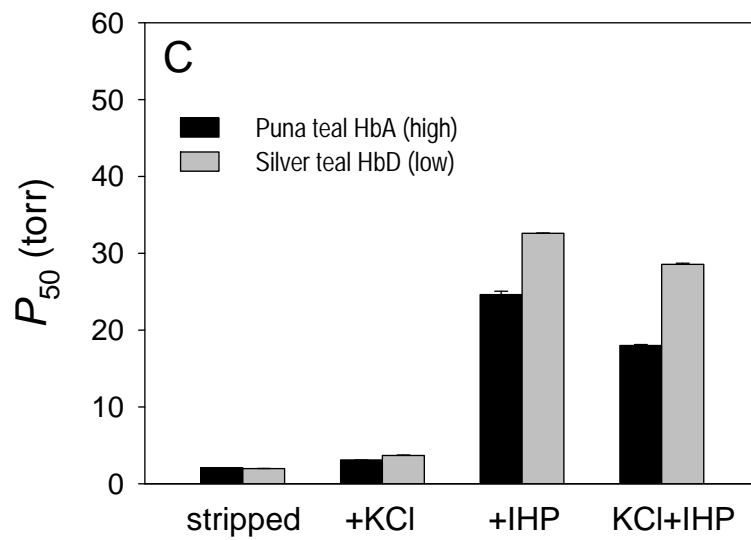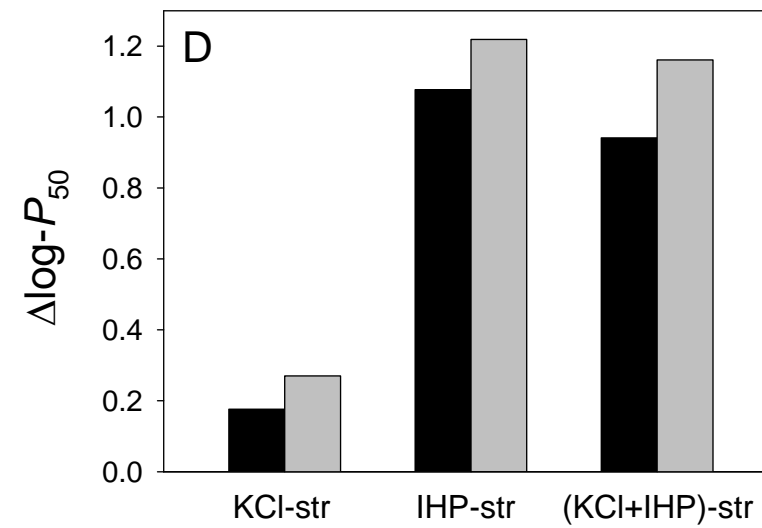

Supplement: S10 Fig — (A) P 50 values (means ± SEM) for purified HbA isoforms of the two species measured at pH 7.4 and 37°C in the absence (stripped) and presence of allosteric effectors ([Cl-], 0.1 M; [HEPES], 0.1 M; IHP/Hb tetramer ratio, 2.0; [Heme], 0.300 mM). (B) Log-transformed differences in P 50 values of HbA isoforms of the two species in the presence and absence of allosteric effectors. The Δlog-P 50 values measure the extent to which Hb-O2 affinity is reduced in the presence of a given allosteric effector (Cl-, IHP, or both anions together). (C) P 50 values for HbD isoforms of Puna teal and silver teal (experimental conditions as above). (D) Δlog-P 50 values for HbD isoforms of the two species. (PDF) [file pgen.1005681.s010.pdf]

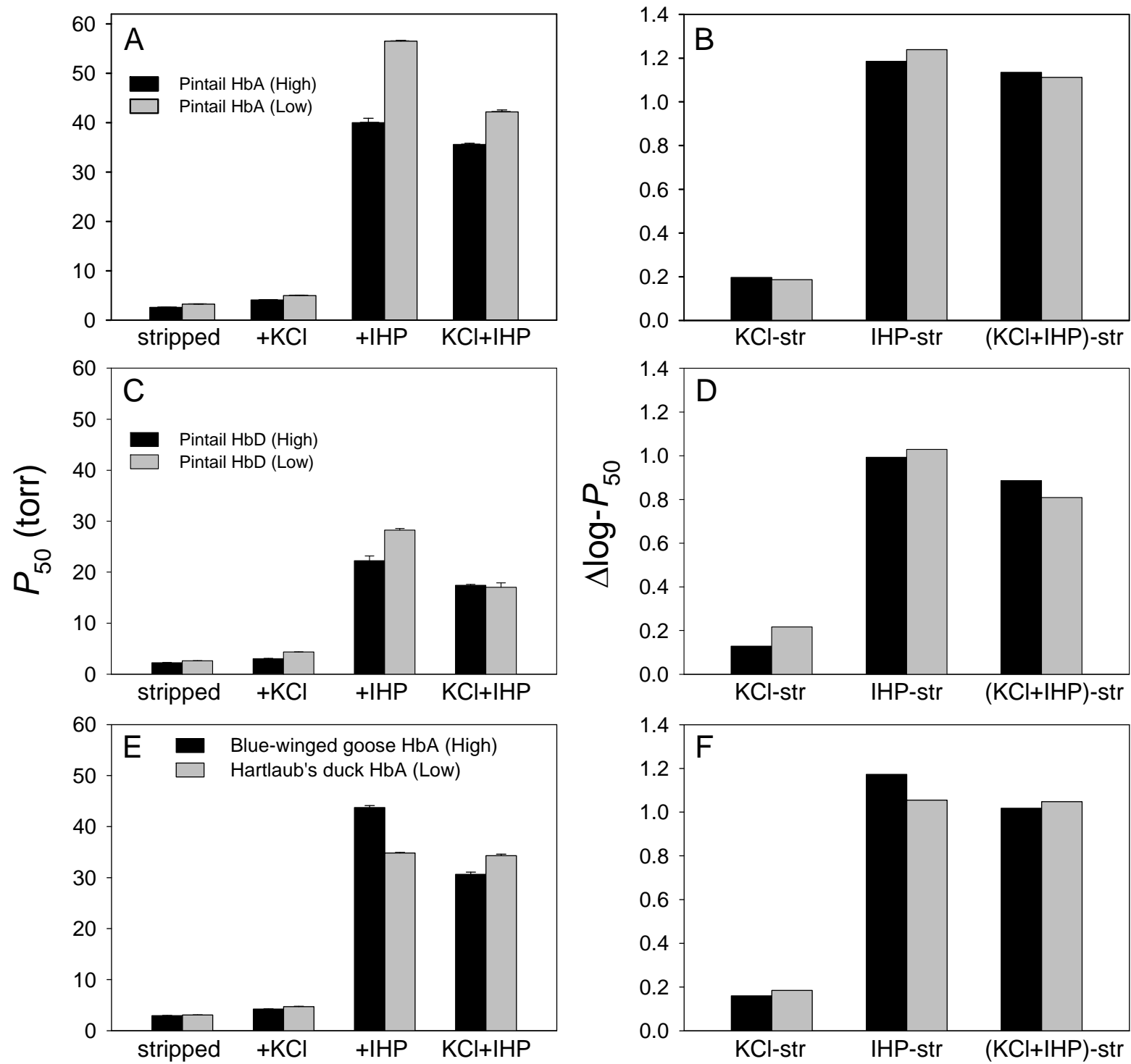

Supplement: S11 Fig — (A) P 50 values (means ± SEM) for purified HbA variants of highland and lowland pintails measured at pH 7.4 and 37°C in the absence (stripped) and presence of allosteric effectors ([Cl-], 0.1 M; [HEPES], 0.1 M; IHP/Hb tetramer ratio, 2.0; [Heme], 0.300 mM). (B) Log-transformed differences in P 50 values of highland and lowland HbA variants in the presence and absence of allosteric effectors. (C) P 50 values for HbD variants of highland and lowland pintails (experimental conditions as above). (D) Δlog-P 50 values for HbD variants of highland and lowland pintails. (E) P 50 values (means ± SEM) for purified HbA isoforms of the blue-winged goose and Hartlaub’s duck (experimental conditions as above). (F) Δlog-P 50 values for HbA isoforms of the blue-winged goose and Hartlaub’s duck. (PDF) [file pgen.1005681.s011.pdf]
