# Supplementary material for: Convergent Evolution of Hemoglobin Function in High-Altitude Andean Waterfowl Involves Limited Parallelism at the Molecular Sequence Level
Source: PLoS Genet. 2015 Dec 4;11(12):e1005681. doi: 10.1371/journal.pgen.1005681 (PMC4670201; doi:10.1371/journal.pgen.1005681)
Supplement: S2 Fig — (PDF) [file pgen.1005681.s002.pdf]

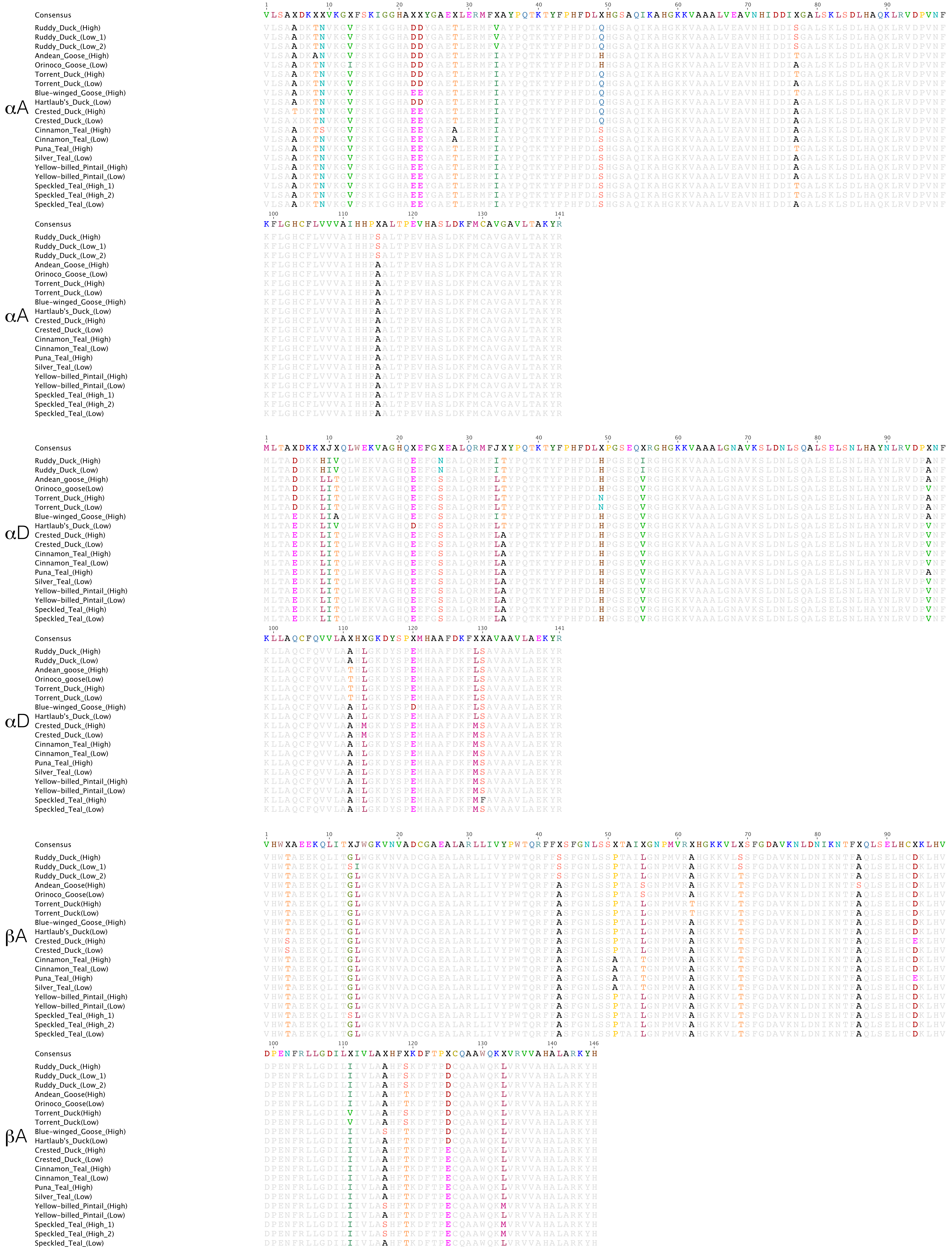
αA
αA
αD
αD
βA
βA
Consensus
Ruddy_Duck_(High)
Ruddy_Duck_(Low_1)
Ruddy_Duck_(Low_2)
Andean_Goose_(High)
Orinoco_Goose_(Low)
Torrent_Duck_(High)
Torrent_Duck_(Low)
Blue-winged_Goose_(High)
Hartlaub's_Duck_(Low)
Crested_Duck_(High)
Crested_Duck_(Low)
Cinnamon_Teal_(High)
Cinnamon_Teal_(Low)
Puna_Teal_(High)
Silver_Teal_(Low)
Yellow-billed_Pintail_(High)
Yellow-billed_Pintail_(Low)
Speckled_Teal_(High_1)
Speckled_Teal_(High_2)
Speckled_Teal_(Low)
