# Supplementary material for: Convergent Evolution of Hemoglobin Function in High-Altitude Andean Waterfowl Involves Limited Parallelism at the Molecular Sequence Level
Source: PLoS Genet. 2015 Dec 4;11(12):e1005681. doi: 10.1371/journal.pgen.1005681 (PMC4670201; doi:10.1371/journal.pgen.1005681)
Supplement: S1 Table — All data are from wild-caught specimens. Sample sizes (no. individuals) are given in parentheses. (DOCX) [file pgen.1005681.s012.docx]

**Table S1**. Percentage concentrations of the HbA and HbD isoforms (mean ± SD [same for both values]) in the red blood cells of high- and low-altitude waterfowl taxa. All data are from wild-caught specimens. Sample sizes (no. individuals) are given in parentheses.

| Taxon | Native altitude | % HbA | % HbD | ± SD |
| --- | --- | --- | --- | --- |
| Ruddy duck (*n* = 9) | High | 81.06 | 18.95 | 0.44 |
| Ruddy duck (*n* = 5) | Low | 81.37 | 18.63 | 0.99 |
| Andean goose (*n* = 7) | High | 81.86 | 18.14 | 2.00 |
| Torrent duck (*n* = 7) | High | 82.39 | 17.61 | 0.98 |
| Torrent duck (*n* = 7) | Low | 82.90 | 17.10 | 0.88 |
| Crested duck (*n* = 8) | High | 74.48 | 25.52 | 2.05 |
| Crested duck (*n* = 8) | Low | 75.62 | 24.38 | 1.49 |
| Cinnamon teal (*n* = 7) | High | 78.05 | 21.95 | 0.76 |
| Cinnamon teal (*n* = 6) | Low | 78.43 | 21.57 | 1.25 |
| Puna teal (*n* = 8) | High | 74.82 | 25.18 | 1.41 |
| Silver teal (*n* = 3) | Low | 73.75 | 26.25 | 0.40 |
| Yellow-billed pintail (*n* = 8) | High | 69.89 | 30.11 | 1.56 |
| Yellow-billed pintail (*n* = 8) | Low | 69.93 | 30.07 | 1.96 |
| Speckled teal (*n* = 7) | High | 70.42 | 29.58 | 1.17 |
| Speckled teal (*n* = 8) | Low | 68.65 | 31.35 | 2.66 |
