# Supplementary material for: Convergent Evolution of Hemoglobin Function in High-Altitude Andean Waterfowl Involves Limited Parallelism at the Molecular Sequence Level
Source: PLoS Genet. 2015 Dec 4;11(12):e1005681. doi: 10.1371/journal.pgen.1005681 (PMC4670201; doi:10.1371/journal.pgen.1005681)
Supplement: S2 Table — O2 equilibria were measured in 0.1 mM HEPES buffer at pH 7.4 (± 0.01) and 37°C in the absence (stripped) and presence of Cl- ions ([KCl]) and IHP (at two-fold molar excess over tetrameric Hb). P 50 and n 50 values were derived from single O2 equilibrium curves, where each value was interpolated from linear Hill plots (correlation coefficient r> 0.995) based on 4 or more equilibrium steps between 25 and 75% saturation. Due to allelic polymorphism, two alternative Hb variants were present in the low-altitude sample of ruddy ducks (‘low1’ and ‘low2’) and in the high-altitude sample of speckled teal (‘high1’ and ‘high2’). In the case of the ruddy ducks, ‘low 1’ and ‘low 2’ represent triply homozygous βA-globin genotypes ‘13Ser-14Ile-69Ser’ and ‘13Gly-14Leu-69Thr’, respectively. In the case of speckled teal, ‘high 1’ and ‘high 2’ represent triply homozygous βA-globin genotypes ‘13Ser-116Ser-133Met’ and ‘13Gly-116Ser-133Met’, respectively. (DOCX) [file pgen.1005681.s013.docx]

**Table S2.** O_2_ affinities (*P*_50_, torr) and cooperativity coefficients (*n*_50_) of purified HbA and HbD isoforms from highland and lowland waterfowl. O_2_ equilibria were measured in 0.1 mM HEPES buffer at pH 7.4 (± 0.01) and 37ºC in the absence (stripped) and presence of Cl^-^ ions ([KCl]) and IHP (at two-fold molar excess over tetrameric Hb). *P*_50_ and *n*_50_ values were derived from single O_2_ equilibrium curves, where each value was interpolated from linear Hill plots (correlation coefficient *r*> 0.995) based on 4 or more equilibrium steps between 25 and 75% saturation. Due to allelic polymorphism, two alternative Hb variants were present in the low-altitude sample of ruddy ducks (‘low1’ and ‘low2’) and in the high-altitude sample of speckled teal (‘high1’ and ‘high2’). In the case of the ruddy ducks, ‘low 1’ and ‘low 2’ represent triply homozygous β*^A^*-globin genotypes ‘13Ser-14Ile-69Ser’ and ‘13Gly-14Leu-69Thr’, respectively. In the case of speckled teal, ‘high 1’ and ‘high 2’ represent triply homozygous β*^A^*-globin genotypes ‘13Ser-116Ser-133Met’ and ‘13Gly-116Ser-133Met’, respectively.

| Species | IsoHb | Stripped | | + KCl | | + IHP | | | | + KCl + IHP | | |  |
| --- | --- | --- | --- | --- | --- | --- | --- | --- | --- | --- | --- | --- | --- |
|  |  | *P*_50_ | *n*_50_ | | *P*_50_ | | *n*_50_ | *P*_50_ | *n*_50_ | | *P*_50_ | *n*_50_ | |
|  |  |  |  | |  | |  |  |  | |  |  | |
| Ruddy duck (high)  Ruddy duck (low1)  Ruddy duck (low2) | HbA  HbD  HbA  HbD  HbA  HbD | 2.84 ± 0.05  2.14 ± 0.05  2.42 ± 0.06  2.38 ± 0.05  3.62 ± 0.07  - | 2.10 ± 0.07  1.75 ± 0.06  1.78 ± 0.08  1.44 ± 0.05  1.55 ± 0.05  - | | 3.98 ± 0.03  2.94 ± 0.08  3.39 ± 0.07  2.84 ± 0.01  4.75 ± 0.17  - | | 2.44 ± 0.04  1.98 ± 0.11  2.00 ± 0.08  1.42 ± 0.01  1.66 ± 0.09  - | 42.11 ± 0.85  29.22 ± 0.17  38.19 ± 1.14  27.76 ± 0.96  42.58 ± 2.47  - | 3.21 ± 0.20  2.93 ± 0.06  2.56 ± 0.18  1.98 ± 0.15  1.38 ± 0.12  - | | 30.05 ± 0.72  20.28 ± 0.34  28.52 ± 0.18  19.34 ± 0.61  28.61 ± 1.56  - | 2.75 ± 0.17  2.55 ± 0.13  2.93 ± 0.06  1.99 ± 0.12  1.83 ± 0.20  - | |
| Andean goose (high)  Orinoco goose (low) | HbA  HbD  HbA  HbD | 2.88 ± 0.07  1.97 ± 0.03  3.14 ± 0.11  2.14 ± 0.03 | 1.79 ± 0.06  1.55 ± 0.03  1.75 ± 0.09  1.62 ± 0.03 | | 4.34 ± 0.13  2.99 ± 0.05  5.31 ± 0.18  3.32 ± 0.06 | | 2.07 ± 0.11  1.84 ± 0.05  1.88 ± 0.09  1.92 ± 0.06 | 34.53 ± 1.10  22.78 ± 0.52  41.17 ± 3.35  22.52 ± 0.87 | 2.30 ± 0.14  2.41 ± 0.12  1.78 ± 0.17  2.21 ± 0.16 | | 27.64 ± 0.92  17.12 ± 0.63  35.31 ± 2.52  20.63 ± 0.84 | 2.41 ± 0.17  2.45 ± 0.20  1.70 ± 0.16  2.46 ± 0.21 | |
| Torrent duck (high) | HbA | 2.01 ± 0.01 | 1.87 ± 0.02 | | 3.03 ± 0.02 | | 2.18 ± 0.03 | 33.45 ± 0.28 | 2.56 ± 0.07 | | 26.60 ± 0.30 | 3.05 ± 0.10 | |
|  | HbD | 1.80 ± 0.03 | 1.27 ± 0.04 | | 2.68 ± 0.02 | | 1.79 ± 0.02 | 21.57 ± 0.06 | 2.64 ± 0.02 | | 17.88 ± 0.16 | 2.92 ± 0.09 | |
| Torrent duck (low) | HbA | 2.42 ± 0.02 | 1.98 ± 0.03 | | 3.48 ± 0.03 | | 2.30 ± 0.04 | 34.95 ± 0.16 | 2.72 ± 0.04 | | 27.97 ± 0.06 | 3.05 ± 0.03 | |
|  | HbD | 1.90 ± 0.02 | 1.50 ± 0.03 | | 2.86 ± 0.04 | | 1.77 ± 0.05 | 22.44 ± 0.24 | 2.48 ± 0.08 | | 18.50 ± 0.01 | 3.08 ± 0.01 | |
| Crested duck (high) | HbA | 2.66 ± 0.02 | 1.54 ± 0.03 | | 4.33 ± 0.03 | | 2.18 ± 0.03 | 34.63 ± 0.05 | 3.07 ± 0.02 | | 25.14 ± 0.25 | 3.01 ± 0.10 | |
|  | HbD | 1.95 ± 0.02 | 1.54 ± 0.04 | | 2.93 ± 0.05 | | 1.83 ± 0.07 | 13.24 ± 0.62 | 1.73 ± 0.19 | | 10.51 ± 0.08 | 2.32 ± 0.06 | |
| Crested duck (low) | HbA | 3.45 ± 0.02 | 2.01 ± 0.03 | | 4.87 ± 0.00 | | 2.22 ± 0.00 | 52.40 ± 0.30 | 2.52 ± 0.04 | | 37.98 ± 0.46 | 2.83 ± 0.12 | |
|  | HbD | 1.93 ± 0.01 | 1.81 ± 0.03 | | 3.16 ± 0.05 | | 1.90 ± 0.07 | 25.90 ± 0.32 | 2.63 ± 0.10 | | 20.35 ± 0.24 | 2.84 ± 0.12 | |
| Cinnamon teal (high) | HbA | 2.37 ± 0.02 | 1.86 ± 0.04 | | 3.59 ± 0.02 | | 2.26 ± 0.04 | 34.79 ± 0.19 | 2.99 ± 0.05 | | 29.36 ± 0.23 | 3.10 ± 0.10 | |
|  | HbD | 2.13 ± 0.01 | 1.84 ± 0.01 | | 3.16 ± 0.06 | | 2.41 ± 0.10 | 36.55 ± 0.36 | 3.49 ± 0.12 | | 26.23 ± 0.10 | 3.45 ± 0.06 | |
| Cinnamon teal (low) | HbA | 3.00 ± 0.03 | 2.06 ± 0.06 | | 4.24 ± 0.05 | | 2.15 ± 0.08 | 47.71 ± 0.35 | 2.79 ± 0.07 | | 37.43 ± 0.13 | 3.04 ± 0.05 | |
|  | HbD | 2.24 ± 0.04 | 1.60 ± 0.05 | | 3.23 ± 0.07 | | 2.02 ± 0.09 | 30.66 ± 0.92 | 2.32 ± 0.17 | | 22.28 ± 0.34 | 2.71 ± 0.12 | |
| Puna teal (high) | HbA | 3.38 ± 0.01 | 2.31 ± 0.75 | | 4.35 ± 0.01 | | 2.18 ± 0.01 | 33.29 ± 0.34 | 2.89 ± 0.11 | | 27.32 ± 0.33 | 2.91 ± 0.11 | |
|  | HbD | 2.06 ± 0.01 | 1.63 ± 0.04 | | 3.09 ± 0.01 | | 1.87 ± 0.01 | 24.61 ± 0.44 | 2.81 ± 0.16 | | 17.98 ± 0.13 | 3.49 ± 0.10 | |
| Silver teal (low) | HbA | 3.93 ± 0.02 | 2.34 ± 0.03 | | 5.80 ± 0.04 | | 2.34 ± 0.05 | 55.55 ± 0.48 | 2.97 ± 0.07 | | 39.66 ± 1.01 | 2.62 ± 0.20 | |
|  | HbD | 1.97 ± 0.01 | 1.81 ± 0.03 | | 3.67 ± 0.06 | | 2.35 ± 0.13 | 32.60 ± 0.03 | 2.84 ± 0.01 | | 28.54 ± 0.15 | 3.12 ± 0.05 | |
| Yellow-billed pintail (high) | HbA | 2.61 ± 0.02 | 1.73 ± 0.02 | | 4.11 ± 0.05 | | 2.25 ± 0.06 | 40.00 ± 0.17 | 3.03 ± 0.06 | | 35.62 ± 0.40 | 2.78 ± 0.09 | |
|  | HbD | 2.26 ± 0.05 | 1.37 ± 0.09 | | 3.04 ± 0.05 | | 1.99 ± 0.08 | 22.25 ± 0.91 | 2.47 ± 0.30 | | 17.41 ± 0.20 | 2.74 ± 0.11 | |
| Yellow-billed pintail (low) | HbA | 3.26 ± 0.03 | 2.13 ± 0.06 | | 5.01 ± 0.07 | | 2.05 ± 0.06 | 56.51 ± 0.91 | 2.41 ± 0.10 | | 42.20 ± 0.23 | 2.77 ± 0.05 | |
|  | HbD | 2.64 ± 0.03 | 2.05 ± 0.08 | | 4.35 ± 0.04 | | 2.55 ± 0.07 | 28.26 ± 0.34 | 3.43 ± 0.14 | | 17.02 ± 0.88 | 1.46 ± 0.18 | |
| Speckled teal (high1) | HbA | 2.14 ± 0.07 | 1.73 ± 0.11 | | 3.48 ± 0.00 | | 2.10 ± 0.00 | 33.91 ± 0.17 | 3.16 ± 0.06 | | 30.08 ± 0.18 | 2.99 ± 0.07 | |
|  | HbD | 1.74 ± 0.02 | 1.36 ± 0.04 | | 2.38 ± 0.05 | | 1.66 ± 0.07 | 18.65 ± 0.09 | 3.18 ± 0.06 | | 13.52 ± 0.13 | 3.18 ± 0.11 | |
| Speckled teal (high2) | HbA | 2.93 ± 0.01 | 2.14 ± 0.03 | | 4.00 ± 0.02 | | 2.23 ± 0.03 | 33.29 ± 0.40 | 3.26 ± 0.15 | | 30.96 ± 0.19 | 3.47 ± 0.09 | |
|  | HbD | 1.57 ± 0.08 | 1.49 ± 0.18 | | 2.73 ± 0.03 | | 1.82 ± 0.05 | 22.00 ± 0.12 | 3.35 ± 0.07 | | 17.64 ± 0.11 | 3.20 ± 0.09 | |
| Speckled teal (low) | HbA | 2.85 ± 0.10 | 1.50 ± 0.14 | | 5.64 ± 0.01 | | 2.53 ± 0.02 | 58.30 ± 0.81 | 2.94 ± 0.19 | | 41.71 ± 0.19 | 3.27 ± 0.08 | |
|  | HbD | 2.87 ± 0.02 | 1.50 ± 0.02 | | 4.10 ± 0.03 | | 2.37 ± 0.04 | 32.28 ± 0.19 | 3.45 ± 0.08 | | 23.81 ± 0.20 | 3.04 ± 0.09 | |
| Blue-winged goose (high) | HbA | 2.94 ± 0.04 | 1.95 ± 0.08 | | 4.25 ± 0.03 | | 2.07 ± 0.05 | 43.72 ± 0.39 | 3.54 ± 0.14 | | 30.64 ± 0.44 | 2.94 ± 0.16 | |
|  | HbD | 2.63 ± 0.03 | 1.72 ± 0.05 | | 4.76 ± 0.02 | | 2.03 ± 0.03 | 21.80 ± 0.34 | 2.61 ± 0.13 | | 21.25 ± 0.55 | 2.41 ± 0.16 | |
| Hartlaub duck (low) | HbA | 3.07 ± 0.03 | 1.97 ± 0.05 | | 4.70 ± 0.09 | | 2.02 ± 0.08 | 34.82 ± 0.11 | 1.81 ± 0.01 | | 34.29 ± 0.30 | 2.38 ± 0.07 | |
|  | HbD | 2.56 ± 0.05 | 1.85 ± 0.09 | | 4.33 ± 0.05 | | 1.99 ± 0.06 | 28.96 ± 0.27 | 3.15 ± 0.12 | | 20.14 ± 0.39 | 2.41 ± 0.17 | |
